# Alternative somatic and germline gene-regulatory strategies during starvation-induced developmental arrest

**DOI:** 10.1016/j.celrep.2022.111473

**Published:** 2022-10-11

**Authors:** Amy K. Webster, Rojin Chitrakar, Seth M. Taylor, L. Ryan Baugh

**Affiliations:** 1 Department of Biology, Duke University, Durham, NC 27708, USA; 2 Center for Genomic and Computational Biology, Duke University, Durham, NC 27708, USA; 3 Present address: Institute of Ecology and Evolution, University of Oregon, Eugene, OR 97403, USA; 4 Lead contact

## Abstract

Nutrient availability governs growth and quiescence, and many animals arrest development when starved. Using *C. elegans* L1 arrest as a model, we show that gene expression changes deep into starvation. Surprisingly, relative expression of germline-enriched genes increases for days. We conditionally degrade the large subunit of RNA polymerase II using the auxin-inducible degron system and analyze absolute expression levels. We find that somatic transcription is required for survival, but the germline maintains transcriptional quiescence. Thousands of genes are continuously transcribed in the soma, though their absolute abundance declines, such that relative expression of germline transcripts increases given extreme transcript stability. Aberrantly activating transcription in starved germ cells compromises reproduction, demonstrating important physiological function of transcriptional quiescence. This work reveals alternative somatic and germline gene-regulatory strategies during starvation, with the soma maintaining a robust transcriptional response to support survival and the germline maintaining transcriptional quiescence to support future reproductive success.

## In brief

Webster et al. show that the transcriptional response to starvation is mounted early in larval somatic cells supporting survival but that it wanes over time. In contrast, they show that the germline remains transcriptionally quiescent deep into starvation, supporting reproductive potential, while maintaining its transcriptome via transcript stability.

## Graphical Abstract

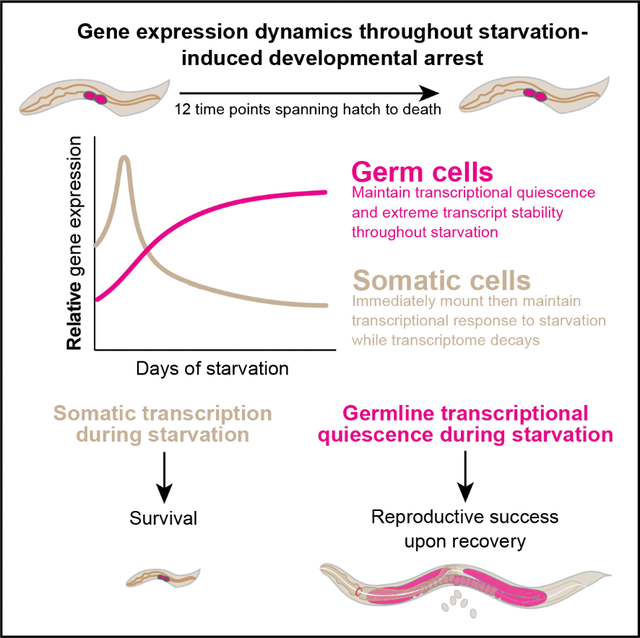

## INTRODUCTION

Development requires favorable environmental conditions, and diverse animals enter a state of developmental arrest in response to unfavorable conditions (MacRae, 2010). Starvation causes cellular quiescence in cells ranging from yeast to human, and some animals arrest development in response to inadequate nutrition (Su et al., 1996; Yao, 2014). *C. elegans* nematodes hatch as L1 larvae, and in the absence of food, they arrest development, providing a valuable model of starvation resistance and developmental arrest (Baugh, 2013). L1 arrest (or L1 diapause) has garnered attention because time spent in arrest does not shorten lifespan upon recovery, as if it is an “ageless” state (Johnson et al., 1984). However, worms in L1 arrest actually exhibit signs of aging, but most are reversible upon recovery (Roux et al., 2016). Nonetheless, extended L1 starvation impacts many life-history traits, including brood size and growth rate (Jobson et al., 2015), and some effects persist across generations (Jobson et al., 2015; Webster et al., 2018). These observations suggest that starvation takes a toll on both somatic and germline cells. These cells have different metabolic demands, developmental constraints, and organismal functions, but how their starvation responses are tailored is unknown.

L1 arrest is accompanied by changes in transcriptional regulation and gene expression. Gene expression profiles of mRNA change rapidly early in L1 arrest (within hours), as the starvation response is mounted (Baugh et al., 2009). A number of transcriptional regulators, including transcription factors, are required to support starvation survival, suggesting a critical role of transcriptional regulation (Baugh and Sternberg, 2006; Zhong et al., 2010; Fukuyama et al., 2012; Baugh, 2013; Cui et al., 2013; O’Rourke and Ruvkun 2013; Kaplan et al., 2015; Murphy et al., 2019; Baugh and Hu, 2020). However, gene expression dynamics that occur beyond 24 h of starvation, and time of action of transcriptional regulation for supporting survival, are largely unknown.

In *C. elegans*, zygotic mRNA transcription begins in somatic blastomeres at the two- to four-cell stage of embryogenesis (Seydoux and Fire, 1994; Baugh et al., 2003), whereas PIE-1 represses transcription in the P lineage, which produces primordial germ cells (PGCs) (Mello et al., 1996; Seydoux et al., 1996; Seydoux and Dunn, 1997). Global repression of transcription in the early embryonic germline is conserved among metazoa, apparently preventing specification of somatic fates (Wang and Seydoux, 2013). Zygotic mRNA transcription begins in PGCs during mid-embryogenesis in *C. elegans* (Wang and Seydoux, 2013). Upon hatching, L1 larvae have 558 cells, two of which are the PGCs, Z2 and Z3. Z2 and Z3 are transcriptionally repressed during L1 arrest, which depends on chromatin compaction genes CEC-4 and HPL-2/HP1 as well as the kinase AMPK and phosphatase DAF-18/PTEN (Demoinet et al., 2017; Belew et al., 2021; Fry et al., 2021). However, the physiological significance of germline transcriptional repression during L1 arrest, and whether this repression is maintained throughout arrest, has not been addressed. It is also unclear if the soma remains transcriptionally active after establishing the starvation response, and the contribution of transcript stability to global gene expression dynamics has not been determined for the soma or germline.

Here, we performed mRNA sequencing (mRNA-seq) on whole, starved L1 larvae over 12 time points spanning the entirety of L1 arrest. We found that gene expression changes throughout starvation, affecting the majority of genes. We used selective degradation of the large subunit of RNA polymerase II (RNA Pol II) AMA-1 in the soma and germline to show that transcription during the first 2 days of starvation in the soma, but at no point in the germline, is required to support starvation survival. Late in starvation, transcription of thousands of genes continues in the soma as a way of maintaining the initial starvation response while the overall amount of mRNA per animal declines. In contrast, the germline is transcriptionally inactive throughout arrest, but germline transcripts are remarkably stable. Critically, disruption of *cec-4* shows that germ cell transcriptional quiescence supports reproductive success upon recovery. Collectively, our results suggest that the soma and germline use distinct regulatory strategies to support organismal fitness during starvation-induced developmental arrest.

## RESULTS

**Figure 1.**
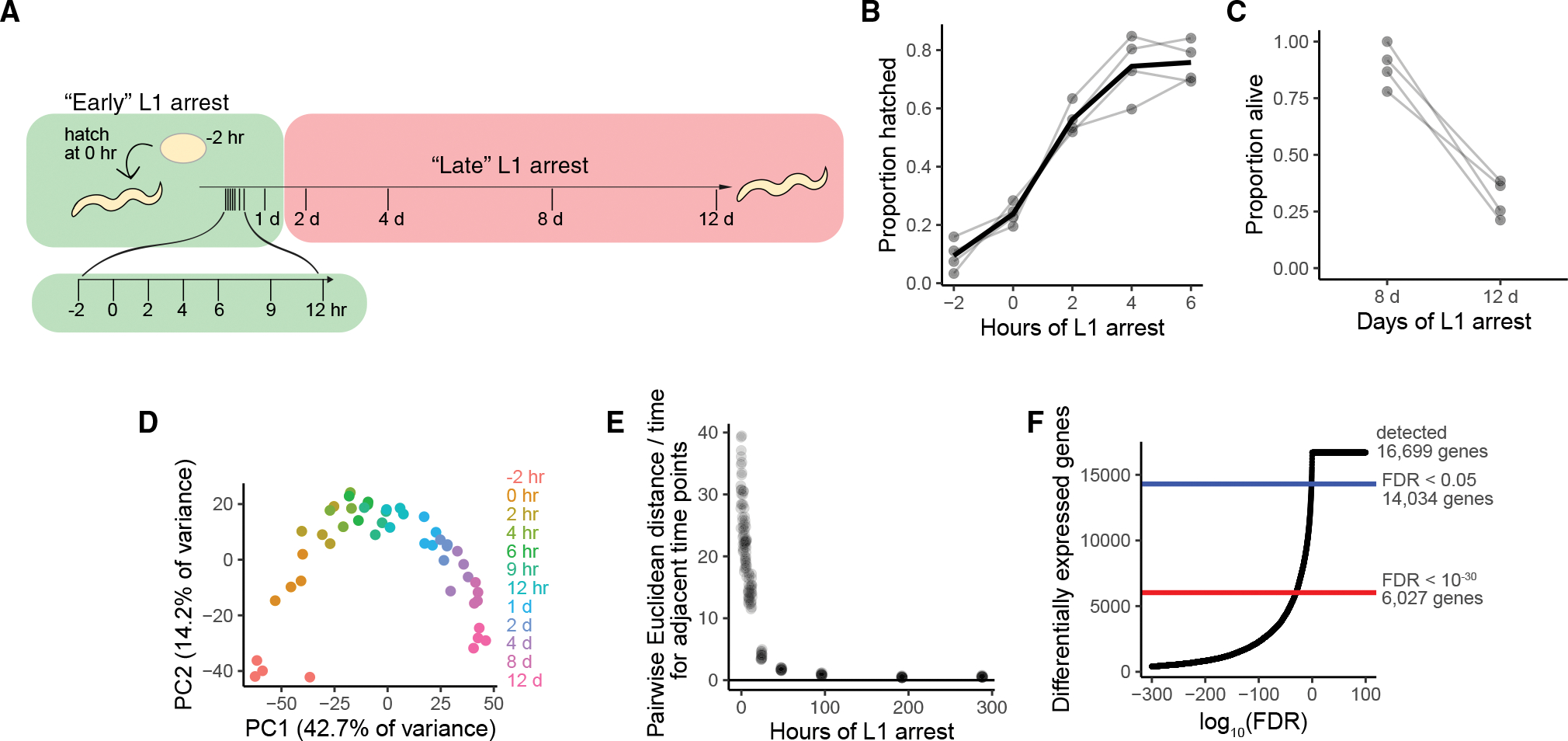
Gene expression changes deep into starvation (A) Time points collected for mRNA-seq time series. (B) Hatching efficiency of all 4 biological replicates at early time points. (C) Survival of all replicates at late time points. (D) Principal-component analysis of RNA-seq data for all time points and replicates. (E) Rate of change of gene expression based on Euclidean distance. (F) Differentially expressed genes as a function of the false discovery rate.

We performed mRNA-seq on whole, starved L1 larvae over time to determine gene expression dynamics throughout L1 arrest. The time series starts approximately 2 h prior to hatching (−2 h) to capture the onset of starvation and extended 12 days beyond that (Figure 1A). Because expression dynamics slow within 12 h (Baugh et al., 2009), we collected time points densely early in arrest and sparsely late in arrest (Figure 1A). ~50% of larvae hatched between 0 and 2 h, and ~80% were hatched by 4 h (Figure 1B), reflecting consistent synchrony and staging. About 80% of larvae were still alive at 8 days, but survival dropped to about 30% at 12 days (Figure 1C). Thus, the time series spans the entirety of L1 arrest, from hatch to death.

### Gene expression dynamics throughout starvation

We performed principal-component analysis (PCA) as an initial evaluation of expression dynamics. Biological replicates clustered together, as expected, and time points were ordered based on the duration of L1 arrest (Figure 1D). A rapid response to starvation was evident in the early hours after hatching, as expected (Baugh et al., 2009). However, time points including day 1 and beyond are clearly distinct from earlier time points, revealing that gene expression continues changing late in starvation. We measured the rate of change between adjacent time points and found that it decreases dramatically during starvation, with a major inflection near 24 h (Figure 1E). These results suggest a rapid early response to starvation followed by a much slower late response extending until death.

Strikingly, the vast majority of genes are differentially expressed during starvation. Over 84% of detected genes (14,034 genes) are differentially expressed at a false discovery rate (FDR) of 0.05, and over 35% (6,027 genes) are differentially expressed at a highly stringent cutoff of 10^−30^ (Figure 1F; Table S1). We generated an RShiny app for users to generate plots of differentially expressed genes of interest over time throughout starvation (https://awebster.shinyapps.io/shinyapp/). This app can also be downloaded and run locally (https://github.com/amykwebster/StarvationTimeSeriesPlots). These results demonstrate the profound effect of starvation on gene expression.

**Figure 2.**
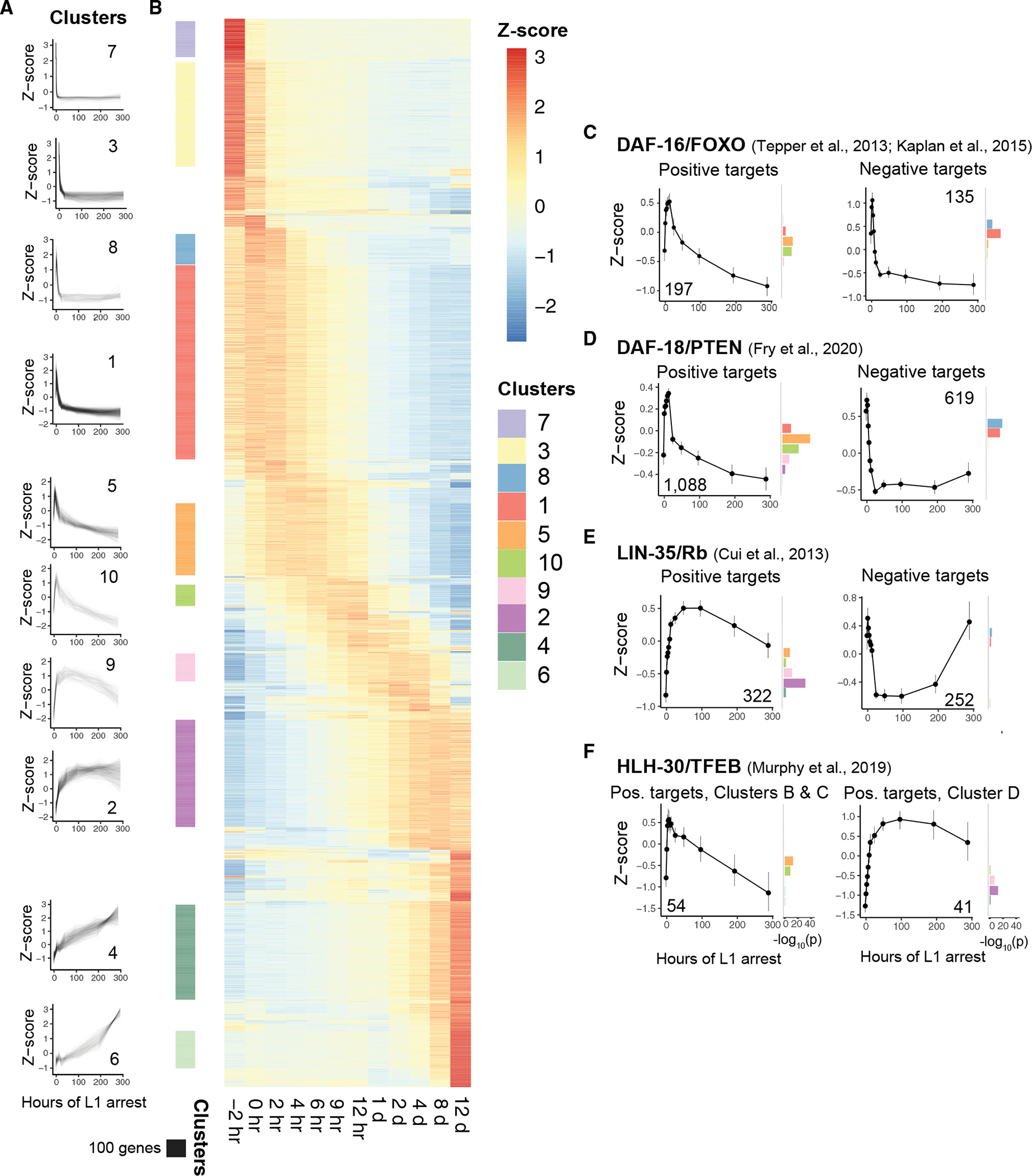
Expression analysis reveals temporal patterns of regulatory activity (A) Z score-normalized expression dynamics over time for the 10 largest clusters. (B) Heatmap of all clustered genes (FDR <10^−30^), sorted by cluster similarity and color coded by *Z* score. Colored bars to the left correspond to genes in clusters shown in (A). (C–F) Gene expression dynamics for known targets of important transcriptional regulators. Number of genes included is inset on each graph. Lines indicate the mean *Z* score and 99% confidence intervals for all genes in each group. To the right of each graph, −log_10_ enrichment p values are plotted for the top 10 clusters.

Despite pervasive effects of starvation, cluster analysis revealed relatively simple temporal patterns. The clustering algorithm used produced 129 clusters for the 6,027 most significantly affected genes, but many of them are distinguished by relatively minor differences in timing or include only a few genes (Figure S1). The 10 largest clusters include about two-thirds of the genes, and these clusters show the predominant expression patterns present in the full dataset. Broadly, these patterns consist of genes monotonically increasing or decreasing in relative expression, even deep into starvation (Figure 2A). The most complex common pattern is an increase followed by a decrease with a single peak early in starvation (e.g., clusters 5, 9, and 10), and other more complex patterns are either very rare or absent (Figures 2B and S1). These observations suggest that the gene-regulatory network controlling the starvation response is relatively shallow compared with developmental regulatory networks.

### Known transcriptional regulators mostly act early in starvation

Previous studies have identified regulators of L1 starvation survival, including transcription factors and signaling molecules that affect transcription factor activity (Baugh and Sternberg, 2006; Zhong et al., 2010; Fukuyama et al., 2012; Baugh, 2013; Cui et al., 2013; O’Rourke and Ruvkun, 2013; Kaplan et al., 2015; Murphy et al., 2019; Baugh and Hu, 2020). We determined expression profiles of known targets (direct and indirect) of critical regulators to shed light on when they are most active. We focused our analysis on DAF-16/FoxO, DAF-18/PTEN, LIN-35/Rb, and HLH-30/TFEB because loss of each severely compromises starvation survival and genome-wide expression data for each mutant in L1 arrest are available (Cui et al., 2013; Tepper et al., 2013; Kaplan et al., 2015; Murphy et al., 2019; Fry et al., 2021). Positively regulated targets (genes down-regulated in the mutant) are expressed at their highest levels at different times after hatching, with DAF-16, DAF-18, and some HLH-30 targets peaking between 6 and 12 h of arrest, and LIN-35 and other HLH-30 targets peaking between 2 and 4 days of L1 arrest (Figures 2C–2F), consistent with relatively early and late function, respectively. SKN-1/Nrf and AMPK targets were identified in later developmental stages without starvation (Steinbaugh et al., 2015; El-Houjeiri et al., 2019), but these targets exhibit peak expression very early in L1 arrest as if these factors help establish the starvation response (Figures S2D and S2E). Like the global dynamics of the starvation response (Figure 1E), these patterns suggest that the transcriptional response to starvation driven by known regulators is largely mounted early. In contrast, regulation accounting for the relative increase in expression observed for hundreds of genes deep in starvation (e.g., clusters 4 and 6) is unknown.

### Differential regulation of germline and somatic genes deep into developmental arrest

**Figure 3.**
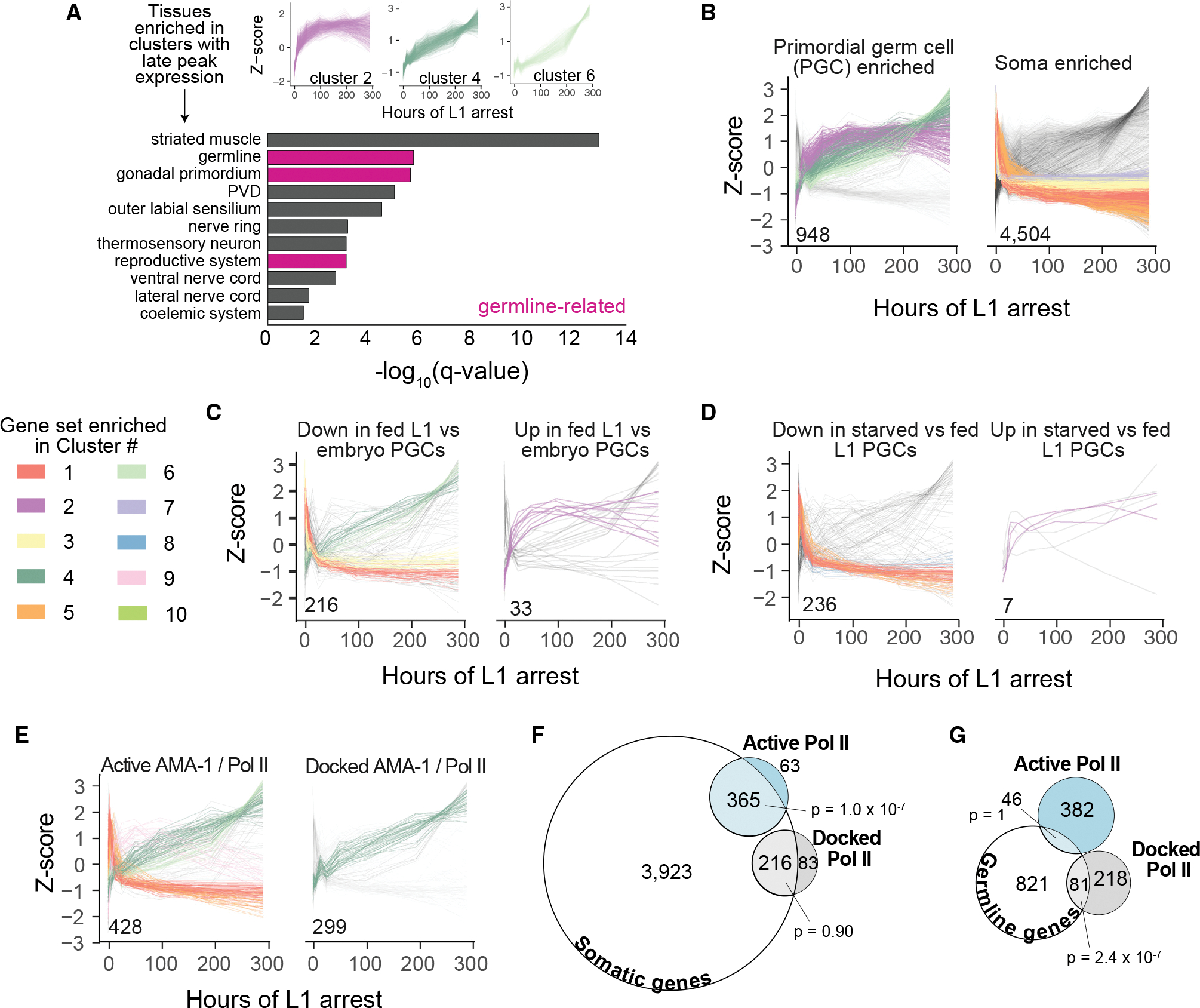
Somatic and germline genes have different patterns of regulation during starvation (A) Tissue enrichments for clusters 2, 4, and 6, which have peak expression late in starvation. Germline-related tissues are highlighted in pink. (B–E) *Z* scores over time are plotted for all individual genes in the indicated group and clustered dataset. The number of genes is inset on each graph. Genes are color coded (see legend) by cluster if the cluster is enriched in the gene group with all other genes in gray. (B and E) Cluster enrichment color coded if hypergeometric p < 0.01. (C and D) Cluster enrichment color coded if hypergeometric p < 0.05. (F) Venn diagram of germline-enriched genes plotted in (B) and genes with active (hypergeometric p = 1) or docked (hypergeometric p = 2.4 × 10^−7^) RNA Pol II. (G) Venn diagram of soma-enriched genes plotted in (B) and genes with active (hypergeometric p = 1.0 × 10^−7^) or docked (hypergeometric p = 0.90) RNA Pol II.

Three of our six largest clusters (clusters 2, 4, and 6) are expressed at relatively low levels upon hatching, exhibit peak expression levels beyond 4 days of starvation, and either maintain or increase expression levels up to 12 days of starvation. These clusters are enriched with genes expressed in several tissues related to reproduction, including “germline,” “gonad primordium,” and “reproductive system” (Figure 3A) (Angeles-Albores et al., 2016), which was surprising given that transcription is repressed in germ cells in the first few hours of L1 arrest (Belew et al., 2021; Fry et al., 2021). We directly assessed if genes typically expressed in the soma or germline exhibit distinct expression patterns. We used published single-cell RNA-seq data from embryos to define gene sets enriched in somatic or PGCs (Packer et al., 2019). PGC-enriched genes are largely expressed at peak levels late in starvation, based on statistical enrichment of clusters 2, 4, and 6 (Figure 3B), and this was robust to defining PGC enrichment with increasing stringency (Figure S3). In contrast, soma-enriched genes are over-represented in clusters with peak expression within the first few hours of starvation (clusters 1, 3, 5, and 7). Collectively, these results suggest that PGC-enriched genes are more likely to increase in relative expression throughout arrest, while soma-enriched genes are more likely to peak early and decrease, though it should be noted that individual genes may deviate from these patterns.

We extended our analysis with a pair of published datasets examining expression in PGCs sorted from L1-stage larvae, including (1) genes differentially expressed between embryonic and fed L1 PGCs and (2) genes differentially expressed between fed and starved L1 PGCs (Lee et al., 2017). As expected, genes down-regulated in fed L1 compared with embryonic PGCs are over-represented in clusters that decrease in expression within hours of hatching (clusters 1 and 3), though they are also enriched in clusters that increase late (clusters 4 and 6) (Figure 3C). In contrast, the majority of genes up-regulated in fed L1 relative to embryonic PGCs are part of the late clusters 2, 4, and 6, with cluster 2 significantly over-represented (Figure 3C). Genes down-regulated in starved compared with fed PGCs are over-represented among clusters with peak expression within the first few hours of L1 arrest (clusters 1, 5, and 8), though the late-peaking cluster 6 is also enriched (Figure 3D). In contrast, six of the seven genes that are up-regulated in starved L1 PGCs exhibit peak expression late in arrest, including three genes significantly enriched in cluster 2 (Figure 3D). Together, these observations suggest that genes with differential expression in L1 PGCs, due to either developmental regulation or starvation, show similar patterns early in L1 arrest in our whole-animal data. They also further support the conclusion that many germline genes increase in relative expression levels deep into starvation.

Distinct temporal patterns of steady-state expression for germline and soma-enriched genes could be driven by active transcription or differences in transcript stability. To gain insight on this distinction, we used previously defined gene sets early in L1 starvation in which RNA Pol II is “active” or “docked” (Maxwell et al., 2014). Active genes accumulate RNA Pol II in the gene body, have evidence of elongation activity and mRNA expression, and are enriched for starvation-response genes. In contrast, docked genes are “poised” in that they accumulate RNA Pol II just upstream of the transcription start site, have little to no elongation activity or mRNA expression, and tend to be immediately up-regulated during recovery from starvation. Genes with active RNA Pol II early in starvation are enriched with clusters with peak expression early, mid-way, and late in starvation (clusters 1 and 5, 9, and 4 and 6, respectively) (Figure 3E). In contrast, genes with docked RNA Pol II are enriched for the late-peaking cluster 4 alone (Figure 3E). We found significant enrichment between differentially expressed somatic genes and active, but not docked, RNA Pol II (Figure 3F), consistent with active transcription of somatic genes early in starvation. We also found significant enrichment between differentially expressed germline-enriched genes and docked, but not active, RNA Pol II (Figure 3G). These associations suggest soma-enriched genes are actively transcribed and germ cells are transcriptional quiescent early in starvation, as expected, but whether the apparent increase of germline and docked gene expression late in starvation is driven by active transcription remains unclear.

### Early somatic, but not germline, transcription supports starvation resistance

**Figure 4.**
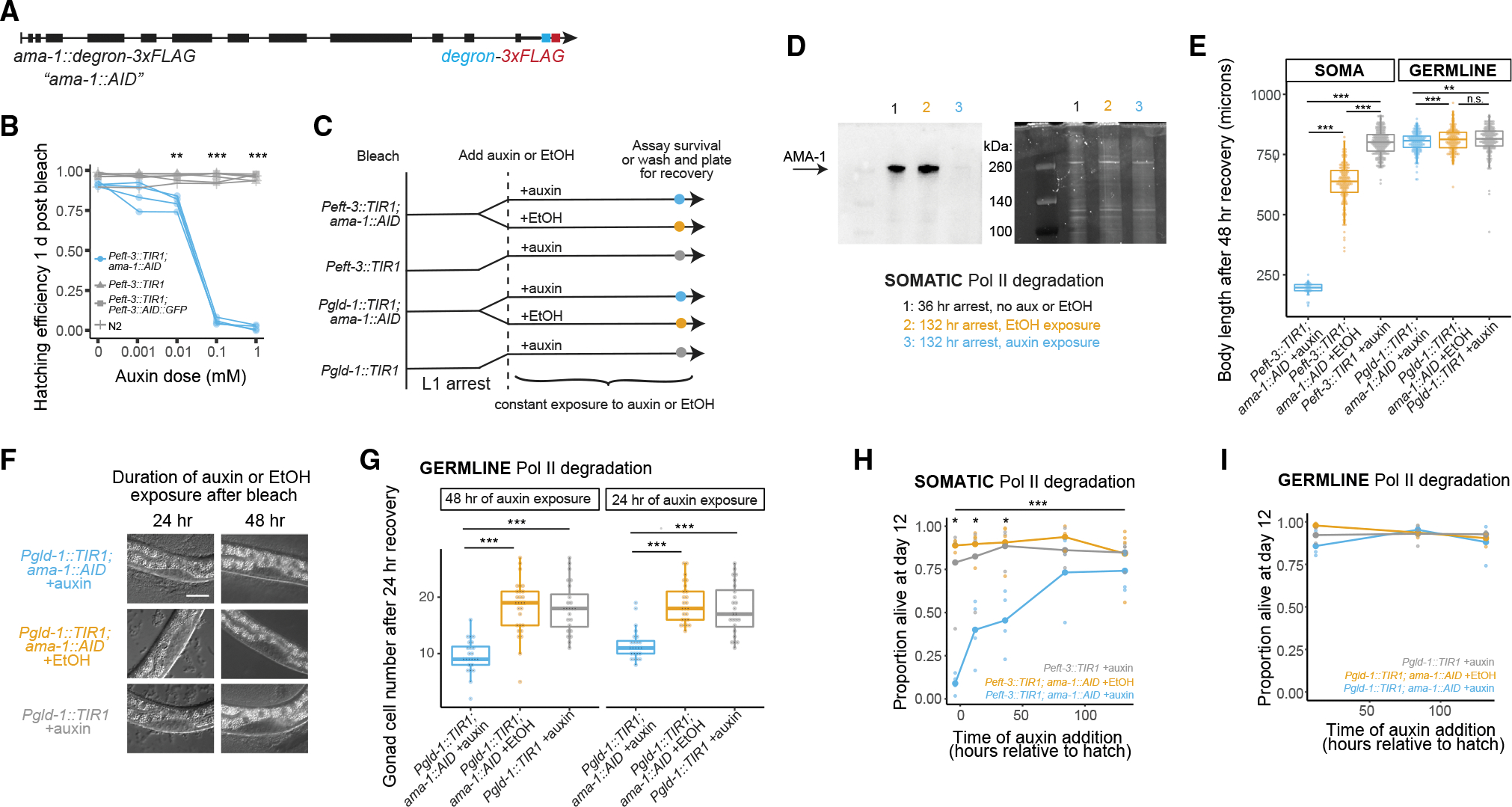
Early somatic transcription supports starvation survival, but germline and late somatic transcription are dispensable (A) Design of *ama-1* degron strain referred to as *ama-1::AID*. The degron and 3x-FLAG sequence were inserted at the C terminus of the endogenous *ama-1* locus. (B) Embryonic hatching efficiency of *Peft-3::TIR1; ama-1::AID* and control strains with different doses of auxin. t tests between *Peft-3::TIR1; ama-1::AID* (n = 4) and each control strain (n = 2) were performed for each dose. ***p < 0.001, **p < 0.01 compared with each control. (C) Experimental design for survival and recovery experiments. Auxin addition to *Peft-3::TIR1; ama-1::AID* degrades AMA-1 in the soma; auxin addition to *Pgld-1::TIR1; ama-1::AID* degrades AMA-1 in the germline. Ethanol (EtOH) addition and auxin addition to *Peft-3::TIR1 and Pgld-1::TIR1* are controls. (D) Western blot of *Peft-3::TIR1; ama-1::AID* after 36 h of arrest without auxin exposure, and with and without auxin exposure at 132 h of arrest. For 132 h samples, auxin or EtOH exposure began at 36 h. Total protein for the corresponding part of the gel is shown to the right. The full western blot and total protein gel including two additional biological replicates are shown in Figure S4. (E) Body length of worms after 48 h recovery with food following exposure of arrested L1s of the indicated strains to auxin or EtOH for 1 h. Linear mixed-effects model with conditions as fixed effect and replicate as random effect. ***p < 0.001, **p < 0.01, n.s., not significant. Points represent individual worms from 3–4 biological replicates. (F) Representative images of the gonadal cells scored in (G). Scale bar indicates 10 μm and applies to all subpanels. (G) Number of gonadal cells in larvae of indicated genotypes recovered with food for 24 h following L1 arrest. Prior to recovery, L1s arrested for 12 h were exposedto 24 h of auxin or EtOH, or embryos immediately post-bleach were exposed to 48 h of auxin or EtOH, both in the absence of food. t tests, ***p < 0.001. Points represent individual worms from 4 biological replicates. (H and I) Survival at 12 days of L1 arrest following constant exposure of indicated genotypes to auxin or EtOH at indicated time point. Pairwise t tests were performed for *Peft-3::TIR1; ama-1::AID* + auxin compared with *Peft-3::TIR1; ama-1::AID* + EtOH and *Peft-3::TIR1* + auxin at each time point. *p < 0.05 in both t tests. One-way ANOVA across all time points for *Peft-3::TIR1; ama-1::AID* + auxin, ***p < 0.001. (H) 4 biological replicates. (I) 3 biological replicates.

To causally ascertain the role of transcription in the soma and germline throughout starvation, we used the auxin-inducible degron (AID) system to selectively degrade the large subunit of RNA Pol II, AMA-1. We used CRISPR to tag AMA-1 with a degron (Figure 4A), and we combined this allele with somatic (*Peft-3::TIR1*) or germline (*Pgld-1::TIR1*) transgenes to enable auxin-dependent degradation of AMA-1 in those tissues (Zhang et al., 2015; Kasimatis et al., 2018). There is a dramatic reduction in hatching efficiency when 0.1 mM auxin is added to *Peft3::TIR1; ama-1::AID* embryos (Figure 4B), consistent with the embryonic arrest caused by transcription inhibition (Edgar et al., 1994; Powell-Coffman et al., 1996). These embryos hatch normally when only solvent is added (ethanol), and wild-type (N2) and *Peft-3::TIR-1* embryos hatch at high frequency when auxin is added (negative controls). When auxin is added to *Pgld-1::TIR1; ama-1::AID* embryos, they develop and hatch, consistent with the germline being dispensable to embryonic development. Post-embryonic development depends on somatic and germline transcription, and auxin treatment causes larval growth arrest in *Peft-3::TIR1; ama-1::AID* worms and sterility in *Pgld-1::TIR1; ama-1::AID* worms (data not shown), as expected. Together, these results suggest that the AID system is effective at selectively degrading AMA-1 in the soma or germline throughout development.

The AID system also effectively eliminates AMA-1 during L1 arrest (Figure 4C). AMA-1 is efficiently degraded in starved L1 larvae when auxin is added to *Peft-3::TIR1; ama-1::AID* (Figures 4D, S4A and S4B), consistent with the AID system in other developmental stages (Zhang et al., 2015) and suggesting a genetic null for *ama-1*. Furthermore, degradation of AMA-1 in the soma during L1 arrest subsequently prevents larval growth during 48 h on food in the absence of auxin (Figure 4E), consistent with somatic transcription being essential to development. Notably, *Peft-3::TIR1; ama-1::AID* worms grow slower in the absence of auxin than *Peft-3::TIR1* worms with auxin. This is consistent with some auxin-independent background degradation of AMA-1, an effect that has been documented for other degron-tagged proteins in the presence of TIR1 (Schiksnis et al., 2020). There is a small but significant effect on body length when auxin is added to *Pgld-1::TIR1; ama-1::AID* worms during L1 arrest, washed thoroughly, and allowed to feed for 48 h (Figure 4E), suggesting germline development is reduced by AMA-1 degradation in the germline during L1 arrest, resulting in smaller worms. To directly address this, we treated *Pgld-1::TIR1; ama-1::AID* embryos and arrested L1s with auxin, incubated them for 48 or 24 h, respectively, washed them thoroughly, recovered them with food for 24 h, and counted the number of gonadal cells (germline and somatic) per worm (Figures 4F and 4G). Germline degradation of AMA-1 during L1 arrest significantly reduces the number of gonadal cells upon recovery to approximately 10–12 per animal on average. Notably, there are approximately 12 somatic gonad cells at the late L2 stage (Hubbard and Greenstein, 2005), suggesting essentially complete inhibition of germ-cell proliferation. Together, these results show that the AID system is effective at selectively degrading AMA-1 in the soma or germline during L1 arrest, and they reveal that doing so inhibits subsequent somatic and germline development, respectively, in fed larvae.

We determined when somatic transcription is essential for starvation survival. We degraded somatic AMA-1 beginning approximately 4 h prior to hatching (−4 h, allowing embryos to hatch and be exposed to auxin for all of L1 arrest), along with 12, 36, 84, and 132 h after hatching without food, and we scored survival on day 12 (~276 h). We chose day 12 because starved worms survive longer in the presence of ethanol (Castro et al., 2012), which is present in auxin and control conditions. Degrading AMA-1 in the soma starting at −4 h severely limits survival (Figure 4H), as expected given early action of critical transcriptional regulators (Figures 2C–2F and S2). Preventing somatic transcription after 12 and 36 h of L1 arrest also reduces survival, but the effect is diminished compared with adding auxin before hatching (−4 h), highlighting the importance of transcription during the first 12 h of L1 arrest. Exposure of wild-type (N2) worms to alpha-amanitin to inhibit transcription starting 12 h after hatching also modestly reduces survival by day 8, corroborating our results in a complementary system (Figure S4C). In contrast, when we degraded AMA-1 starting at 84 and 132 h, we did not detect a significant decrease in survival, suggesting that late transcription is less important for survival. These results demonstrate that somatic transcription is initially required to mount the starvation response but that transcription is largely dispensable after that, consistent with the observed inflection in gene expression dynamics during early L1 arrest (Figure 1E).

We determined if germline transcription supports starvation survival. (Figure 4I). We added auxin to *Peft-3::TIR1; ama-1::AID* worms 12, 84, and 132 h after hatching without food. The later treatments allowed us to determine whether late transcription of germline genes, a possibility suggested by the time series, impacts survival positively or negatively. However, degradation of germline AMA-1 does not affect survival. This suggests that germline transcription, if it occurs, is dispensable for starvation survival, though it is required for recovery (Figures 4F and 4G).

### Transcription occurs in the soma, but not germline, days into starvation

**Figure 5.**
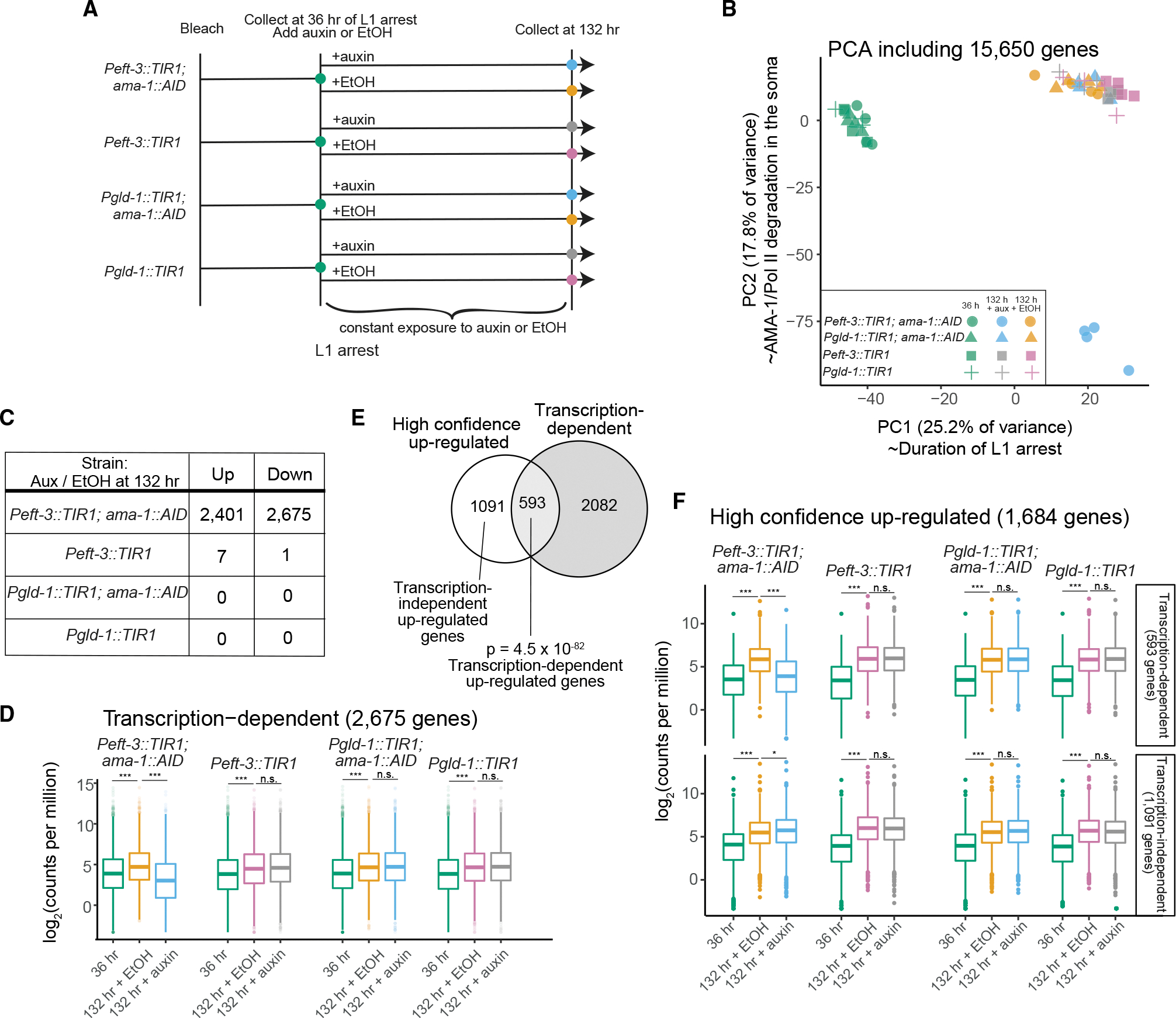
Transcription-dependent gene expression changes occur in the soma, but not the germline, late in starvation (A) Experimental design of mRNA-seq experiment. Arrested L1s of indicated genotypes are collected at 36 h of arrest. After constant exposure to auxin or EtOH from 36 h onward, arrested L1s of indicated genotypes are collected at 132 h of arrest. 4 biological replicates were sequenced for each condition and time point. (B) Principal-component analysis of all conditions and replicates. (C) Number of differentially expressed genes for each genotype at 132 h of L1 arrest depending on whether they were exposed to auxin or EtOH. (D) Log_2_ counts per million plotted for all transcription-dependent genes (genes down-regulated in *Peft-3::TIR1; ama-1::AID* + auxin compared with *Peft-3::TIR1; ama-1::AID* + EtOH) across all conditions. (E) Venn diagram of “high-confidence up-regulated genes” (genes up-regulated at 132 h with EtOH exposure compared with 36 h in all four genotypes) and “transcription-dependent genes.” Hypergeometric p value is shown. (F) Log_2_ counts per million reads plotted for all “high-confidence up-regulated genes,” parsed by whether they are transcription dependent or transcription independent, as designated in (E). (D and F) Significance determined using the Kolmogorov-Smirnov test. In each strain background, 36 h versus 132 h + EtOH and 132 h + EtOH versus 132 h + auxin were compared. *p < 0.05, **p < 0.01, ***p < 0.001.

We next sought to determine whether gene expression changes days into starvation are driven by ongoing transcription or differences in transcript stability. We performed an additional mRNA-seq experiment with samples collected after 36 or 132 h of L1 arrest, with auxin or ethanol added at 36 h, and *Peft-3::TIR1* and *Pgld-1::TIR1* strains were included as controls (Figure 5A). Worms had not died due to lack of transcription at 132 h (Figure S5A), facilitating collection of RNA. PCA revealed that time spent in arrest explains more variance in the data than any other factor, with clear separation of 36- and 132-h samples in principal component 1 (Figure 5B). Principal component 2 separates samples with somatic AMA-1 degraded from all others, demonstrating that transcription plays a role in shaping late expression dynamics. Notably, we did not see separation of samples with germline AMA-1 degraded, suggesting relatively little transcription in germ cells, consistent with our survival results (Figure 4I). In addition, all controls cluster together, suggesting that addition of auxin to *Peft-3::TIR1* or *Pgld-1::TIR* alone does not substantially affect gene expression. We identified differentially expressed genes for each strain at 132 h by comparing auxin and ethanol treatments. For *Peft-3::TIR1; ama-1::AID* and *Pgld-1::TIR1; ama-1::AID* strains, differentially expressed genes indicate transcription-dependent gene expression changes in the soma or germline, respectively. Using an FDR of 0.05, the results corroborate PCA, identifying thousands of genes with increased or decreased transcript abundance when somatic AMA-1 is degraded, zero genes when germline AMA-1 is degraded, and almost no genes in the control strains (Figure 5C; Table S1). Because mRNA-seq measures relative transcript abundance (i.e., counts per million), it is unlikely that genes that increase in transcript abundance with AMA-1 degradation are increased in absolute terms. When a large proportion of genes are changing, as in our datasets, changes in transcript abundance should be considered relative (Risso et al., 2014; Evans et al., 2018). We conclude that somatic transcription plays a substantial role in shaping gene-expression dynamics late in L1 arrest and that germ cells remain transcriptionally quiescent deep into arrest.

We were particularly interested in the 2,675 genes with decreased expression when somatic AMA-1 was degraded. On average, these “transcription-dependent” genes are relatively up-regulated between 36 and 132 h of arrest with ethanol in all four strains (Figure 5D). This temporal increase in expression is abrogated when somatic AMA-1 is degraded but not when germline AMA-1 is degraded or in control strains. Again, these results suggest a role of somatic, but not germline, transcription beyond 36 h of arrest.

We determined the proportion of genes with increased expression over time due to transcription. 1,684 genes are consistently up-regulated between 36 and 132 h with ethanol in all four strains, and 35% of these “high-confidence up-regulated” genes are also among the 2,675 transcription-dependent genes (Figure 5E). We refer to this overlapping set as “transcription-dependent up-regulated genes.” On average, this gene set behaves like the larger transcription-dependent set (Figure 5D), with an increase in expression over time in all cases except when somatic AMA-1 is degraded, though the effect sizes are larger for the transcription-dependent up-regulated genes (Figure 5F). These results show that transcription drives increased expression of these genes late in arrest. In contrast, high confidence up-regulated genes that are not also classified as transcription-dependent (“transcription-independent up-regulated genes”) have increased average expression over time even when somatic AMA-1 is degraded, consistent with greater relative transcript stability driving increased relative expression of these genes. We conclude that transcription is the primary cause of increased expression late in starvation for about one-third of up-regulated genes but that differences in transcript stability drive increases in relative expression for about two-thirds of apparently up-regulated genes.

### Increased stability of germline genes compared with somatic genes

**Figure 6.**
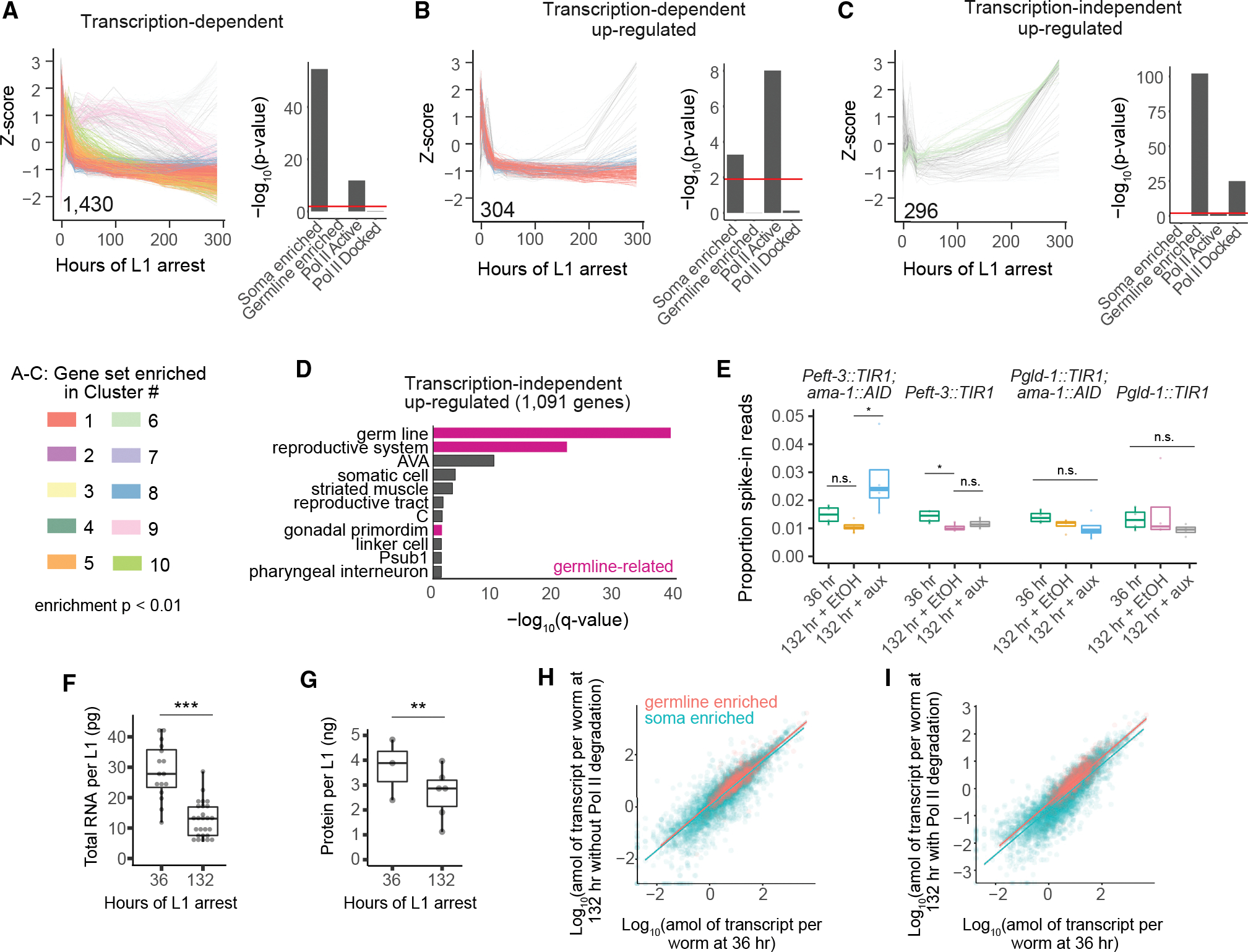
Transcription-independent increased expression late in starvation is driven by relative stability of germline transcripts (A–C) *Z* score over time is plotted for all individual genes in the indicated gene group that are part of the clustering dataset. The number of genes plotted is inset on each graph. Genes are color coded by cluster if that cluster is enriched (hypergeometric p < 0.01) in the gene group. Hypergeometric p values are plotted for each gene group and their overlap with soma-enriched, germline-enriched, RNA Pol II active, and RNA Pol II docked genes (shown in Figure 3). (D) Tissue enrichments of all “high-confidence up-regulated genes.” Germline tissues are highlighted in pink. (E) Proportion of reads mapping to spike-in transcripts from all 4 biological replicates for each condition. One-way ANOVAs across three conditions in each genetic background were performed. If p < 0.05, then pairwise t tests were performed. *p ≤ 0.05 in t test. (F) Total RNA for all available samples used in RNA-seq separated by duration of starvation with all genotypes and conditions included. Points are individual samples from 4 biological replicates. (G) Total protein for *Peft3::TIR1; ama-1::AID* samples from 3 biological replicates included in western blot. (F–G) Linear mixed-effects model with duration of starvation as a fixed effect and biological replicate as a random effect, **p < 0.01, ***p < 0.001. (H and I) Log_10_-transformed attomoles of transcript per worm calculated based on spike-ins and total RNA yield per worm for somatic and germline transcripts in the *ama-1::AID; Peft-3::TIR1* strain at 36 and 132 h with EtOH (no degradation of AMA-1) (H) or auxin (degradation of AMA-1) (I). Lines indicate linear regression for each gene group with line width indicating 95% confidence interval.

In our original time series, we found that differentially expressed soma-enriched genes exhibit peak expression in the first hours of arrest and are associated with active RNA Pol II, while differentially expressed germ-cell-enriched genes exhibit peak expression days into arrest and are associated with docked RNA Pol II (Figure 3). Strikingly, transcription-dependent genes are most strongly enriched for clusters with peak expression early in arrest (clusters 1, 5, 8, 9, and 10) (Figure 6A). Notably, clusters with increasing expression late in arrest (e.g., clusters 2, 4, and 6) are not enriched, nor are clusters with peak expression in late embryos (clusters 3 and 7). As a subset of transcription-dependent genes, transcription-dependent up-regulated genes are enriched among clusters with peak expression early in arrest (clusters 1 and 8) (Figure 6B). Both gene sets are enriched for somatic genes and RNA Pol II active genes but not germline genes or RNA Pol II docked genes. Thus, transcription-dependent genes are transcribed late in arrest as a continuation of the early starvation response, even though the majority of them do not increase expression late in arrest. In contrast, transcription-independent up-regulated genes are enriched for cluster 6, which increases in relative expression late in arrest (Figure 6C). Furthermore, they are enriched for germline genes and RNA Pol II docked genes. In addition, the germline and reproductive system are highly enriched among transcription-independent up-regulated genes (Figure 6D), and the significance of enrichment is substantially greater than for all genes in clusters with late peak expression (Figure 3A). These results support the conclusion that greater relative transcript stability drives late increases among germline-enriched genes rather than transcription.

As a further check that germline-enriched genes are relatively stable compared with soma-enriched genes, we used external standards for absolute quantification of mRNA abundance. During library preparation for the AMA-1 AID mRNA-seq experiment (Figure 5A), we added a pool of 92 synthetic transcripts (spike-ins) at known concentrations based on total RNA content. Because a constant spike-in-to-total RNA ratio was used, determining the proportion of mRNA-seq reads mapping to spike-ins provides a proxy for mRNA content, with a higher proportion mapping to spike-ins indicating a lower mRNA-to-total RNA ratio. *Peft-3::TIR1; ama-1::AID* worms at 132 h with auxin has a significantly higher proportion of reads mapping to spike-ins compared with the ethanol control, showing that, consistent with expectation, mRNA decreases relative to total RNA when AMA-1 is degraded in the soma (Figure 6E). There is not a significant increase in the proportion of spike-in reads between 36 and 132 h with ethanol in any strain background (Figure 6E), suggesting a relatively constant mRNA-to-total RNA ratio is maintained between 36 and 132 h. We assessed total RNA levels for samples used in the mRNA-seq experiment. At 132 h, there is a significant, approximately two-fold decrease in total RNA isolated relative to 36 h, with RNA isolated from equal numbers of larvae (Figures 6F and S5). Together, these observations suggest that total RNA declines late in starvation and that mRNA declines approximately in tandem. In addition to a decline in total RNA, total protein declined between 36 and 132 h (Figures 6G and S5), and larvae shrink throughout L1 arrest (Hibshman, 2017), consistent with a systemic decline in macromolecular content per individual.

Using total RNA and spike-ins to normalize mRNA-seq count data to transcripts per worm, we found that absolute expression levels of germline-enriched genes are more resistant to AMA-1 degradation than soma-enriched genes (Figures 6H and 6I). Both somatic and germline-enriched transcripts decline upon AMA-1 degradation between 36 and 132 h, but this decline is larger for soma-enriched genes. These results support the conclusion that germline-enriched transcripts are particularly stable, maintaining their relative expression levels deep into starvation despite lack of germline transcription. This is in contrast to soma-enriched genes, which continue to be transcribed as their transcript abundance nonetheless wanes.

### Germline transcriptional quiescence during starvation supports reproductive potential

**Figure 7.**
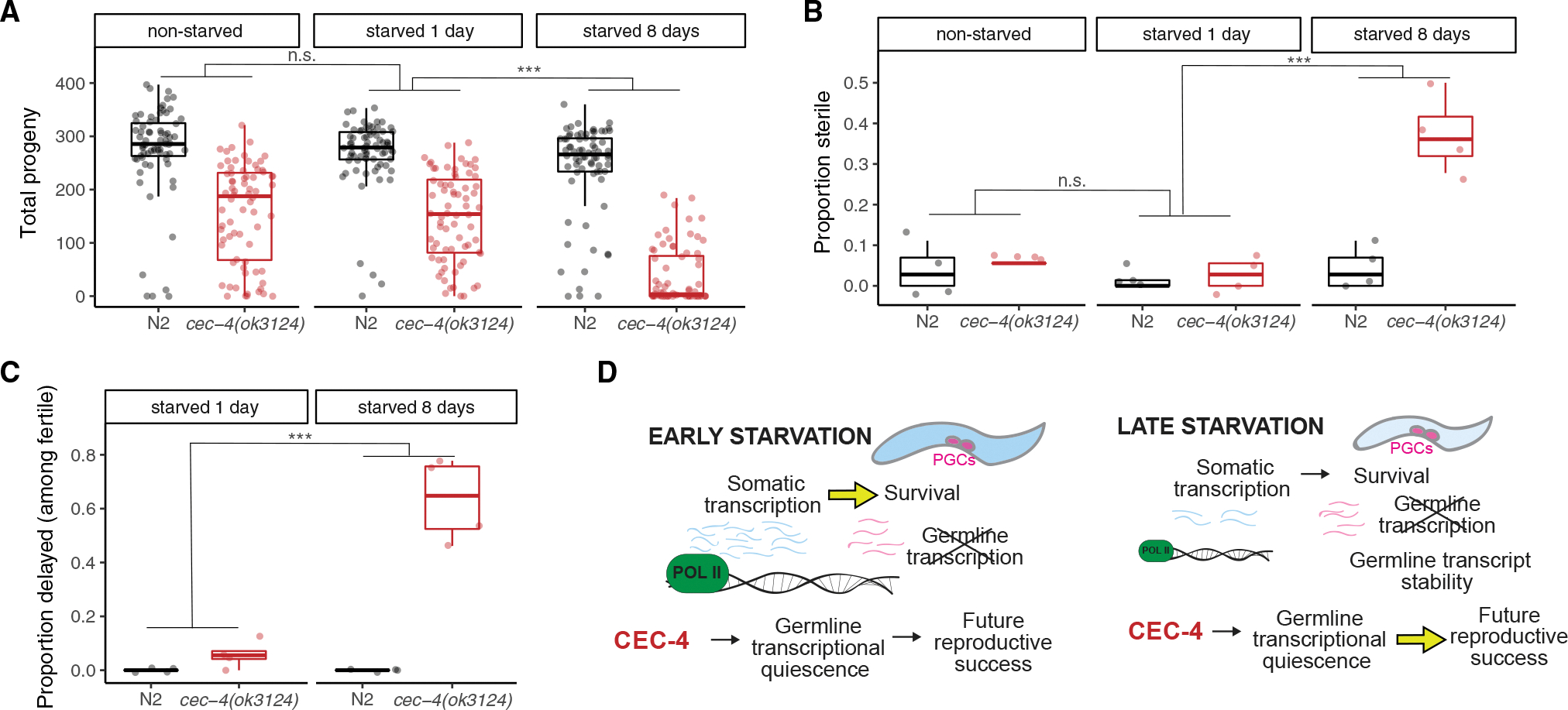
Transcriptional quiescence is required in germ cells deep in starvation to promote reproductive success upon recovery (A) Total brood size of N2 and *cec-4(ok3124)* worms either without starvation, after 1 day of L1 starvation, or after 8 days of L1 starvation. 4 biological replicates, 18 worms in each biological replicate, strain, and day combination. (B) Proportion of worms assayed for brood size in (A) that are completely sterile (total brood = 0). Each point represents a biological replicate. (C) Among fertile worms (total brood >0), proportion of worms with delayed egg-laying onset, which is considered past 72 h on feeding following arrest. Data are shown for worms that experienced starvation (1 or 8 days), but not non-starved worms, because starved worms are developmentally synchronized by plating on food. (A–C) Linear mixed-effect model interaction p value between 0 and 1 days of starvation or between 1 and 8 days of starvation. n.s., not significant (p > 0.05), ***p < 0.001. (D) Summary of somatic and germline gene regulation during starvation-induced developmental arrest (see discussion).

We were interested in assessing the physiological significance of germline transcriptional quiescence during L1 arrest. Belew et al. recently identified CEC-4, a chromodomain protein involved in chromatin compaction, as essential for such quiescence (Belew et al., 2021). However, it is not known if germline transcriptional quiescence supports developmental arrest during starvation or fitness upon recovery. We analyzed a putative null mutant, *cec-4(ok314),* shown by Belew et al. to disrupt PGC chromatin compaction. We found that the mutant PGCs do not proliferate during L1 starvation (data not shown), suggesting that transcriptional activation is not sufficient for PGC proliferation, though it is necessary (Fry et al., 2021). However, the mutant has reduced brood size overall, and the effect is particularly pronounced following 8 days of L1 starvation (Figure 7A), revealing a significant consequence of transcriptional activation in starved PGCs. In addition, after 8 days of starvation (but not 0 or 1 day), *cec-4(ok3134)* mutants are significantly more likely to be sterile (with a total brood of zero) (Figure 7B). Further, even among fertile worms (at least one progeny produced), the majority of *cec-4(ok3124)* mutant worms are delayed in reaching egg-laying onset after 8 days of starvation, an effect that is not apparent after only 1 day of starvation (Figure 7C). Collectively, this analysis reveals substantial fitness consequences to worms that are unable to maintain germline transcriptional quiescence deep into L1 arrest.

## DISCUSSION

We used mRNA-seq to characterize gene expression dynamics in whole L1-stage *C. elegans* larvae throughout starvation-induced developmental arrest. We leveraged a variety of genome-scale datasets for integrative analysis of our results, allowing us to infer mechanisms and anatomical sites of regulation. We also generated an easy-to-use resource for users to plot gene expression for genes of interest over time (https://awebster.shinyapps.io/shinyapp/, https://github.com/amykwebster/StarvationTimeSeriesPlots). We took advantage of the AID system to conditionally degrade AMA-1/RNA Pol II in the soma or germline, assessing the role of transcription in shaping expression dynamics during starvation and its contribution to survival. Together, our results suggest a model (Figure 7D) in which early somatic transcription is essential to mount the starvation response in support of survival, and ongoing somatic transcription maintains this response, but late transcription does not appreciably support survival. In contrast to the soma, the germline remains largely transcriptionally quiescent throughout arrest, but germline transcripts are stable, while somatic transcripts turn over and collectively decline during arrest, leading to a relative increase of germline transcript abundance late in arrest. Finally, we assessed the physiological significance of germline transcriptional quiescence, finding that it supports reproductive success of arrested larvae upon recovery.

### Gene expression continues changing deep into starvation-induced developmental arrest

Gene expression analysis of starvation and developmental arrest typically focuses on a relatively early phase of the response (Wang and Kim, 2003; Baugh et al., 2009; Sinha et al., 2012). Such work has revealed pervasive effects of nutrient availability on gene expression, but expression dynamics deep into cellular quiescence and developmental arrest have not been characterized. Our time-series analysis reveals a rapid, widespread response within hours of hatching in the absence of food followed by a gradual but dramatic decline in the rate of transcriptome change. Nonetheless, gene expression levels continue changing throughout starvation, with a few thousand genes displaying increases in relative transcript abundance in whole worms deep into starvation. Notably, ~84% of detected genes are differentially expressed over time during starvation, highlighting the vast physiological changes that take place.

Gene set enrichment analysis revealed differences in temporal expression patterns and regulation of genes with expression biased toward the soma or germline. We used multiple published datasets that specify genes with expression in germ or somatic cells (Angeles-Albores et al., 2016, 2018; Lee et al., 2017; Packer et al., 2019) to associate tissues or cell types with temporal expression patterns. Surprisingly, many genes expressed in the germline display peak expression levels deep into starvation. This is generally in contrast to genes expressed in the soma, whose expression tends to peak early. However, genes expressed in muscle are a notable exception in that they also tend to peak late. We also found that genes with a poised form of RNA Pol II (docked RNA Pol II) (Maxwell et al., 2014) are enriched among genes expressed in the germline and genes whose expression peaks late. Again, this is in contrast to soma-expressed genes, which are instead associated with an active, elongating form of RNA Pol II. These intriguing patterns of germline gene expression and regulation raise important questions. For example, why are genes with an inactive, docked form of RNA Pol II increasing in expression late into starvation, and why are germline-expressed genes displaying this pattern in particular?

### Somatic transcription is required early in arrest to promote starvation survival

The dramatic and essentially immediate gene expression response to starvation suggests a critical role of early transcription. Indeed, genes regulated by DAF-16/FoxO, DAF-18/PTEN, AMPK, LIN-35/Rb, and HLH-30/TFEB, each of which contributes to transcriptional regulation and is essential to starvation survival, are differentially expressed within hours of hatching without food. These results are consistent with a critical role of early transcription in mounting the starvation response, and DAF-16/FoxO nuclear localization dynamics support this interpretation (Weinkove et al., 2006; Mata-Cabana et al., 2020). To go beyond correlation, we used the AID system to conditionally degrade AMA-1/RNA Pol II starting at different times in L1 arrest. Critically, transcription is required relatively early to support survival days later, with its requirement easing between 36 and 84 h. The transcriptional inhibitor alpha-amanitin corroborates these findings. These results functionally demonstrate the importance of transcription in initially mounting the starvation response. mRNA-seq, together with AMA-1/RNA Pol II degradation, revealed ongoing transcription of thousands of genes late in arrest, but expression of these genes peaks early, as if their ongoing transcription reflects maintenance of the starvation response. In contrast to developmental gene-regulatory networks, which produce expression cascades, the lack of a distinct late starvation response suggests a relatively shallow regulatory network. Notably, degradation of AMA-1/RNA Pol II with tissue-specific TIR1 transgenes revealed that somatic transcription promotes starvation survival but that germline transcription does not affect survival. These results are consistent with transcription deploying and actively maintaining the starvation response in somatic tissues while the germline is transcriptionally quiescent (see below).

### The germline remains transcriptionally quiescent throughout developmental arrest

Since it was a surprising observation, we focused on the apparent up-regulation of germline-expressed genes late in starvation. The germline is transcriptionally quiescent for much of embryogenesis (Seydoux et al., 1996; Seydoux and Dunn, 1997; Wang and Seydoux, 2013), and bulk zygotic genome activation (ZGA) occurs in PGCs in response to feeding in L1 larvae (Furuhashi et al., 2010; Butuci et al., 2015). Global chromatin compaction and transcriptional repression occur in the PGCs of larvae that hatch without food and enter L1 arrest (Belew et al., 2021). Such compaction depends on the chromodomain protein CEC-4, the heterochromatin protein HPL-2/HP1, and the energy-sensing kinase AMPK (Belew et al., 2021), and AMPK mutants have abnormally high levels of chromatin marks for transcriptional activation in PGCs of starved L1 larvae (Demoinet et al., 2017). The tumor suppressor DAF-18/PTEN is also required to inhibit PGC transcription in starved L1 larvae (Fry et al., 2021). In trying to understand the apparent up-regulation of germline-expressed genes late in starvation, we wondered if germline ZGA occurs deep into L1 arrest, as if transcriptional repression eventually fails in wild type as it does in some mutants. However, by eliminating transcription and measuring absolute transcript abundance genome wide, we found that germline genes are not transcribed during extended starvation. This work extends on previous findings (Belew et al., 2021; Fry et al., 2021) by showing that transcriptional quiescence in the germline is sufficiently robust to last for days (rather than hours). Furthermore, by analyzing a *cec-4* mutant, which permits activation of transcription in starved PGCs, we show that transcriptional quiescence supports future reproductive success with presumed fitness consequences for the animal. Transcriptional quiescence of germline cells, and possibly other stem cells, during starvation is likely conserved among metazoa.

### Transcript stability maintains germline gene expression deep into starvation

mRNA-seq measures steady-state mRNA expression levels, and differential expression may be due to differences in transcription, transcript stability, or both. Many studies assume that up-regulation is due to transcription, which is a reasonable assumption if the expression levels of most genes are not changing. However, it is difficult to know at the outset of an experiment whether this is the case, and notable exceptions exist (Radonjic et al., 2005; Lin et al., 2012). We found that germline-expressed genes increase relative expression late in arrest regardless of whether RNA Pol II is present. This suggests that apparent up-regulation of germline genes is driven by transcript stability rather than transcription. Notably, expression of these genes is enriched in germ cells, but it is not exclusive to germ cells, and some somatic transcripts may also be exceptionally stable. In addition to using the AID system to conditionally eliminate transcription in the soma or germline, we used 92 synthetic transcripts at known concentrations as spike-in standards with mRNA-seq. This approach enabled us to infer the relative fraction of total RNA represented by polyadenylated mRNA in each sample, and it allowed us to convert read counts to absolute estimates of transcript abundance per worm. Total RNA per worm declines deep into starvation, and the mRNA-to-total RNA ratio remains approximately constant, suggesting germline genes remain relatively stable as the overall transcriptome declines, accounting for their apparent up-regulation in whole worms. Notably, we also found that protein levels decrease during L1 arrest, and we previously reported that arrested L1s shrink (Hibshman, 2017), consistent with autophagy and other processes supporting survival but contributing to somatic collapse as starved larvae approach death. Together these approaches demonstrate that the amount of mRNA per worm decreases deep into starvation and that germline-enriched transcripts are generally more stable than those enriched in the soma.

Blocking transcription genetically or pharmacologically is a common way to measure the stability of transcripts in yeast (Herruer et al., 1988; Wang et al., 2002), and interpretation of relative up-regulation as increased stability when transcription is inhibited has precedent (Grigull et al., 2004). This interpretation is also corroborated by the association of germline genes with docked, but not active, RNA Pol II. Eukaryotic mRNA half-lives can range from minutes to days (Lugowski et al., 2018), and mRNA half-lives can be increased in response to stress (Hollams et al., 2002). In our study, adequate mRNA was available for generating RNA-seq libraries with a standard protocol for at least 4 days after RNA Pol II degradation, suggesting exceptional transcript stability in a starved, developmentally arrested state. Some studies have linked transcript degradation to translation (Radhakrishnan and Green, 2016). We speculate that transcripts important for germ cell identity and/or initiation of PGC proliferation (such as maternal germline transcripts) tend not to be translated during starvation, allowing them to more easily escape degradation than transcripts important for survival.

### Limitations of this study

This study analyzes mRNA expression in whole worms, and it focuses on differences between somatic and germline cells. However, somatic and germline expression are inferred from other studies that utilized cell sorting or single-cell RNA-seq. Ideally, this study would have used one or both of these methods, though neither method has been used with such complex experimental designs (i.e., in a time series, with disruption of transcription, or with absolute quantification of transcript abundance). In addition, there are substantial differences between somatic cell types, though we largely consider them in aggregate. Genes expressed in striated muscle exhibit similar dynamics to germline-enriched genes, and investigation of underlying regulatory mechanism would be of interest. Finally, we report that germline transcripts are remarkably stable during starvation, but we have not investigated the physiological significance of this observation. We speculate that transcript stability supports germline identity and development upon recovery from starvation, but we were not able to identify a perturbation that would allow us to test this hypothesis.

## STAR★METHODS

### RESOURCE AVAILABILITY

#### Lead contact

Further information and requests for resources and reagents should be directed to and will be fulfilled by the lead contact, Ryan Baugh (ryan.baugh@duke.edu).

#### Materials availability

Strains generated in this study are available upon request.

#### Data and code availability

mRNA-seq datasets generated in this study are available at NCBI GEO at GSE173657. Plots of genes of interest during starvation can also be plotted with an online tool (https://awebster.shinyapps.io/shinyapp/), or the tool can be downloaded and run locally (https://github.com/amykwebster/StarvationTimeSeriesPlots). Code used for mRNA-seq analysis and to generate figures is archived on Zenodo (https://doi.org/10.5281/zenodo.7063257). Any additional information required to reanalyze the data reported in this paper is available from the lead contact upon request.

### EXPERIMENTAL MODEL AND SUBJECT DETAILS

N2 was obtained from the Sternberg collection at the California Institute of Technology, originally from the CGC in 1987. Strains obtained from the CGC are:

CA1200 ieSi57 [*eft-3p*::TIR1::mRuby::*unc-54* 3′UTR + Cbr-*unc*-*119*(+)] II – referred to as *Peft-3::TIR1*

CA1202 ieSi57 II; ieSi58 [*eft-3p*::degron::GFP::*unc-54* 3′UTR + Cbr-*unc-119*(+)] IV – referred to as *Peft-3::TIR1; Peft-3::AID::GFP*

CA1352 ieSi64 [*gld-1p*::TIR1::mRuby::*gld-1* 3′UTR + Cbr-*unc-119*(+)] II – referred to as *Pgld-1::TIR1* RB2301 *cec-4(ok3124)* IV

Newly generated strains from this study are:

PHX1513 ama-1(syb1513) – referred to as ama-1::AID

LRB387 ieSi64 II; ama-1(syb1513) IV – referred to as Pgld-1::TIR1; ama-1::AID

LRB389 ieSi57 II; ama-1(syb1513) IV – referred to as Peft-3::TIR1; ama-1::AID

### METHOD DETAILS

#### Sample preparation and collection for mRNA-seq time series

N2 was maintained well-fed on OP50 at 20°C for at least three generations. For each biological replicate, seven to ten gravid adults were picked onto approximately 20 large (10 cm) NGM plates with OP50 3.5 days prior to hypochlorite treatment (bleach). Large plates were then hypochlorite treated as described previously (Hibshman et al., 2021) to obtain over 200,000 embryos per biological replicate. Embryos were resuspended in S-basal at a density of 1 embryo/μL in an Erlenmeyer flask at 20°C in a shaker at 180 rpm. Beginning at 10 h after hypochlorite treatment, hatching efficiency was scored. Time points used in the time series are 12 h offset from hypochlorite treatment and signify when worms have begun hatching and are thus undergoing L1 arrest. L1 larvae hatch approximately 12 h after hypochlorite treatment, and this is considered 0 h. Scoring continued every two hours until the curve appeared to level off at 18 h after hypochlorite treatment, or 6 h of L1 arrest (Figure 1B). At least 10,000 L1s were collected per time point. For days 8 and 12, the number of L1s collected was doubled and quadrupled, respectively, to account for lethality at later time points. To collect samples, L1s in S-basal were first spun down at 3,000 rpm, and S-basal was aspirated off down to less than 100 μL. The pellet and residual S-basal was transferred to a 1.5 mL Eppendorf tube using a glass pipet. Samples were flash frozen in liquid nitrogen and stored at −80°C until RNA isolation.

#### RNA isolation and library preparation for mRNA-seq time series

RNA was extracted using TRIzol Reagent (Thermo-Fisher Scientific) using the manufacturer’s protocol with some exceptions. 100 μL of sand (Sigma-Aldrich) was included to aid homogenization. Sand was first prepared by washing two times in 1 M HCl, washed eight times in RNAse-free water (to a neutral pH), and baked to dry. Libraries were prepared for sequencing using the NEBNext Ultra RNA Library Prep Kit for Illumina (New England Biolabs, E7530) with 100 ng of starting RNA per library and 14 cycles of PCR. Libraries were sequenced using Illumina HiSeq 4000. Four biological replicates were sequenced per time point. Three replicates consisted of samples collected from the same culture (12 time points collected from a single culture). One replicate consisted of samples from multiple original cultures.

#### Differential expression analysis for mRNA-seq time series

Single-end 50 bp reads were mapped with with bowtie (Langmead 2010) using the following command: bowtie –best –chunkmbs 256 -k 1 -m 2 -S -p 2. Average mapping efficiency was 81.5% with a standard deviation of 2.0%. HTseq was used to count reads mapping to genes (Anders et al., 2015) using version WS210 of the *C. elegans* genome obtained from Maxwell et al. (Maxwell et al., 2012). Count tables were used to detect differential expression in R using edgeR (Robinson et al., 2010). Prior to differential expression analysis, count tables were filtered first to include only genes with counts per million (CPM) greater than 1 across four libraries. This resulted in 16,699 genes for further analysis. The edgeR glm functionality was used to fit a GLM, and a likelihood ratio test was performed for each gene; this ANOVA-like test was used to detect any differences across the twelve time points. PCA was done on log2 mean-normalized CPM values for the 16,699 detected genes (Figure 1D). For rate-of-change analysis, the Euclidean distance between all replicates for adjacent time points (using the same input as for PCA) was calculated and divided by the duration of time between the time points (Figure 1E).

#### Clustering and heatmap of time series

6,027 genes with FDR <1 × 10^−30^ were included in cluster analysis. Using the twelve average CPM values over time for each gene, the pairwise Pearson correlation coefficient was calculated for each gene compared to every other gene. 1 minus the Pearson correlation coefficient was calculated, and these values were assembled into a square matrix to use as input for clustering. Clustering was performed in Matlab, using a published algorithm (Heyer et al., 1999; Baugh et al., 2003). As parameters for clustering, 0.2 was used as the maximum distance for two genes to still be called in the same cluster, where the “distance” is again defined as 1 minus the Pearson correlation coefficient. Thus, genes in the same cluster have at minimum a Pearson correlation coefficient of 0.8. This non-deterministic analysis resulted in 129 clusters, with approximately two-thirds of genes in the top 10 clusters and smaller clusters containing as few as two genes.

To plot genes within clusters, the Z-score for each gene over the 12 time points was calculated. The Z-score scales expression by the mean and standard deviation rather than abundance. Plots were generated for each cluster in R using the package ggplot2. To generate heatmaps, the R package pheatmap was used. 6,027 genes used for clustering were plotted in the heatmap. Genes within the same cluster were plotted next to each other without additional sorting. Clusters were first sorted by time point of highest average expression assessed by Z-score. Clusters with the same time point of highest expression were sorted by their ‘centroid’ time point. The centroid was calculated by transforming z-scores for each cluster so that the minimum was 0, calculating the sum across all 12 time points, and calculating the first time point at which the sum of the transformed Z-scores is less than half of the total sum. For clusters with the same maximum and centroid time point, clusters were sorted based on the value of the maximum Z-score. Clusters with peak expression at earlier time points were plotted before those at later time points. Among clusters with the same peak expression time point, clusters were ordered by the time point. For clusters with peak expression at the same time point, clusters were ordered based on the Z-score at the peak. Clusters were ordered from highest to lowest for peaks in the first six time points, and from lowest to highest for peaks in the last six time points.

#### Gene enrichment analysis for gene groups of interest

Gene groups were either obtained from datasets in previous publications (including those used in Figures 2C–2F and 3) or generated as part of this study (Figure 6). For RNAseq datasets, genes down-regulated in a transcriptional regulator mutant relative to wild-type were considered positive targets, and genes up-regulated were considered negative targets. Wormbase IDs were used for clustered genes and gene groups of interest. If Wormbase IDs were not included in the original publication’s dataset, then Simplemine was used to convert gene identifiers. Gene groups were filtered to include only genes that were considered expressed in the time series dataset. For each cluster, the overlap between the filtered gene group and the cluster’s genes was determined, and a hypergeometric p value was calculated. For plots including the mean Z-score over time with 99% confidence intervals (*i.e*., Figure 2), the stat_summary function in ggplot2 was used, and bootstrapping (mean_cl_boot) was the method for determining the confidence interval surrounding the mean. For plots including individual trajectories for all genes in the filtered gene group included in clustering (*i.e*. Figures 3 and 6), lines were color-coded if the gene was part of an enriched cluster. Clusters were considered enriched if p < 0.01 or p < 0.05, as indicated in the figure legend. A more stringent p value was used with larger gene groups. Genes in non-enriched clusters were still plotted, but with less opacity to enable visualization of all gene trajectories.

#### Design and generation of ama-1 AID strains

We designed PHX1513 *ama-1(syb1513)* to have an insertion prior to the stop codon at the endogenous *ama-1* locus. This insertion contained the degron sequence, linkers, and 3x-FLAG tag, in line with designs used to create other AID strains in *C. elegans* (Zhang et al., 2015; Kasimatis et al., 2018). The sequence of the insertion used was:

GGATCCGGAGGTGGCGGGATGCCTAAAGATCCAGCCAAACCTCCGGCCAAGGCACAAGTTGTGGGATGGCCACCGGTGAGATCATACCGGAAGAACGTGATGGTTTCCTGCCAAAAATCAAGCGGTGGCCCGGAGGCGGCGGCGTTCGTGAAGGAGAATCTGTACTTTCAATCCGGAAAGGACTACAAAGACCATGACGGTGATTATAAAGATCATGATATCGATTACAAGGATGACGATGACAAGTAA

SunyBiotech generated the strain PHX1513 and validated it with Sanger sequencing. We also validated the strain upon receipt using Sanger sequencing. To generate the functional AID strains LRB387 and LRB389, PHX1513 was backcrossed twice to N2 and then crossed to strains expressing TIR1 in either the soma (CA1200) or germline (CA1352).

#### Auxin preparation and treatment

A 400 mM stock solution of indole-3-acetic acid (IAA), or auxin, was prepared in ethanol and stored at −20°C. For experiments using a 0.1 mM auxin dose, a 100 mM stock in ethanol was used, and a corresponding amount of ethanol was used (0.1%) for the control. For experiments using a 1 mM auxin dose, the 400 mM stock was used, and a corresponding amount of ethanol (0.25%) was used for the control.

#### Starvation survival assays

Starvation survival in Figure 1C was assayed by indirect scoring (Baugh and Hu 2020), which considers worms alive if they are able to recover upon feeding, as done previously (Webster et al., 2018). 100 μL of each 8-day and 12-day sample used for RNA-seq was aliquoted around the edge of a 5 cm NGM plate with a spot of OP50 in the center, and the number of individuals plated was counted (~100 worms). Survivors were scored two days later, and proportion alive was calculated.

For Figures 4H and 4I, direct scoring in arrested L1s was used to assay survival because worms with degraded AMA-1 in the soma could not recover. To prepare plates to bleach, ~10 L4s from *Peft-3::TIR1, Pgld-1::TIR1*, and *Pgld-1::TIR1; ama-1::AID* were picked onto approximately four 10 cm plates with OP50. ~15 adults were picked from *Peft-3::TIR1; ama-1::AID* onto at least seven 10 cm plates with OP50 to account for slower growth and fewer progeny from this strain. 4 days later, gravid adults from each genotype were hypochlorite treated from unstarved plates. S-basal with 0.1% ethanol was used throughout washing and hypochlorite treatment. For each condition (genotype and auxin or ethanol addition at each time point), a 5 mL culture of S-basal with 0.1% ethanol was set up in a 25 mL Erlenmeyer flask at 1 embryo/μL (5,000 embryos total) and stored in a 20°C shaker moving at 180 rpm. At the indicated time point, 0.1 mM auxin was added for + auxin conditions, and 0.1% ethanol was added for ethanol conditions. This ensured an equal amount of ethanol (final concentration of 0.2%) across all conditions. After 12 days of L1 arrest, at least 100 μL (~100 worms) was sampled from the culture and pipetted on to a glass depression slide. Worms were scored as dead if they were rods or lacked muscle tone and had a granular appearance without apparent movement. Proportion alive was calculated as the number of live worms divided by the sum of alive and dead worms. For Figure 4H, a minimum of 59 worms were counted per condition and replicate, with an average of 94 worms across all conditions and replicates. For Figure 4I, a minimum of 71 worms were counted per condition and replicate, with an average of 108 across all conditions and replicates. All raw data can be found in Table S2.

For starvation survival with alpha-amanitin (Figure S4C), direct scoring was also used. N2 worms were hypochlorite treated and embryos were resuspended in S-basal at 1 embryo/μL in Erlenmeyer flasks and placed in a shaker, as was done with the *ama-1::AID* strains. 12 h after hatch (24 h after hypochlorite treatment), 25 ug/mL alpha-amanitin was added to cultures. Survival was scored on days 2 and 8, with day 8 as an earlier last time point than with AMA-1 AID because ethanol was not added to the cultures. A minimum of 73 worms were counted for each condition and replicate, with an average of 114 across all conditions and replicates. All raw data can be found in Table S2.

#### Starvation recovery

For Figure 4E, strains were scaled up and hypochlorite treated as described in ‘starvation survival assays,’ but without ethanol added to S-basal. Embryos were resuspended at 1 embryo/μL in 5 mL of S-basal in a 16 mm glass test tube and stored on a roller drum at 20°C. After 23 h (11 h of L1 arrest), 1 mM auxin or 0.25% ethanol was added to each test tube. After 1 h, worms were transferred to 15 mL conical tubes and spun down at 3,000 rpm, and excess S-basal was aspirated off. S-basal was used to wash worms three additional times, then they were plated on 10 cm NGM plates with OP50. After 48 h of growth, worms from each condition were washed off of plates with S-basal, spun down, and transferred to an unseeded 10 cm NGM plate for imaging. Images were taken on a ZeissDiscovery.V20 stereomicroscope and analyzed using the WormSizer plugin for FIJI (Moore et al., 2013). A total of 1,092 worms were scored across all replicates and conditions. For all conditions except *Peft-3::TIR1; ama-1::AID* + auxin, at least 31 worms were scored for each replicate. Because *Peft-3::TIR1; ama-1::AID* + auxin did not recover, however, accurate measurements of larvae were obtained for 41 worms across 4 replicates. All raw data is available as part of Table S2.

#### Total brood size, sterility, and egg-laying onset

For Figures 7A–7C, *cec-4(ok3124)* and N2 worms starved 1 or 8 days were starved as described for starvation survival assays in virgin S-basal (no cholesterol or ethanol), then plated on NGM plates with OP50 following starvation. For non-starved worms, embryos were immediately plated on NGM plates with OP50 following bleach. After 48 h on OP50 (60 h for non-starved worms to account for embryonic development), 18 worms for each strain, replicate, and time point were singled to individual OP50 plates, then transferred to new plates every 24 h until egg-laying ceased. Approximately 2 days after removal of worms from a plate, the progeny laid on that plate were counted. Total brood sizes for each worm were determined by summing the number of progeny laid on each day. If the total brood size was zero, the worm was considered sterile. The proportion of sterile worms was calculated for each replicate, strain, and time point as shown in Figure 7B. Delayed worms (Figure 7C) were those that did not commence egg-laying in the first day (hours 48–72) but did later lay at least 1 embryo. Statistics for 7A-C were performed with linear mixed-effect models, using strain and days of starvation as fixed effects, and the biological replicate as a random effect. A p value was calculated for the interaction between the number of days of starvation (0 vs. 1 or 1 vs. 8) and the strain (N2 or *cec-4(ok3124)*). Raw data is available in Table S2.

#### Hatching efficiency

5 cm NGM plates with OP50 were prepared with different doses of auxin (1, 0.1, 0.01, 0.001, 0.0001 mM) by pipetting the appropriate amount of auxin and ethanol onto an already-seeded plate immediately before use. A cell spreader was used to quickly and lightly (to minimize interfering with the lawn) spread the auxin on the plate. Doses of auxin were prepared from the 400 mM auxin stock, and lower doses were diluted in ethanol so that the same amount of ethanol was used for each dose. Embryos resuspended in S-basal following hypochlorite treatment were pipetted onto the plates prepared for each dose. After 24 h, the number of embryos and L1s were scored on each plate. 100–300 individual animals were scored for each replicate and condition, and raw data is available as part of Table S2.

#### Gonad cell scoring upon recovery

Approximately 10 *Peft-3::TIR1* and *Peft-3::TIR1; ama-1::AID* L4s were picked onto each of 3–4 10 cm NGM plates seeded with OP50. 4 days later, strains were hypochlorite treated as described for ‘starvation survival assays’, with S-basal with 0.1% ethanol included throughout. 5 mL cultures were set up with embryos resuspended at 1 embryo/μL in 16 mm glass test tubes. Immediately after cultures were set up (within approximately 1 h post-bleach), 0.1 mM auxin or 0.1% ethanol was added to cultures for the 48 h exposure conditions. Test tubes were then put on a rotating roller drum at 20°C. At 24 h post-bleach (approximately 12 h of L1 arrest), 0.1 mM auxin or 0.1% ethanol was added to cultures for the 24-h exposure conditions. At 48 h post-bleach, all six cultures (both 24-h and 48-h exposures) were transferred to 15 mL conical tubes, spun down at 3,000 rpm, and excess S-basal was aspirated off down to <100 μL containing the arrested L1s. Worms were washed six times with 10 mL S-basal. After all washes, worms were transferred to 10 cm NGM plates seeded with OP50. After 24 h of growth on plates, 4% agar slides were prepared for viewing on a compound microscope. 2 μL of levamisole was pipetted onto each slide, individual worms were picked from plates into the levamisole, and a coverslip was placed. Individual cells in the gonad were then counted using an AxioImager compound microscope (Zeiss) at 1000x total magnification. DIC images were taken using Zen software (Zeiss), and cropped in Adobe Illustrator. 8–11 worms were scored for each condition and replicate, and all raw data is available in Table S2.

#### Sample preparation and collection for mRNA-seq on AMA-1 AID samples

All strains were maintained well-fed on OP50 at 20°C for several generations. For *Pgld-1;:TIR1; ama-1::AID, Peft-3::TIR1*, and *Pgld-1::TIR1,* approximately 10 L4s were picked onto five 10 cm NGM plates with OP50 for each biological replicate. For *Peft-3::TIR1; ama-1::AID*, which grows slower, approximately 15 gravid adults were picked onto seven 10 cm NGM plates with OP50 for each biological replicate. After 4 days, plates from all four strains were hypochlorite treated in parallel. For each strain, three 50 mL Erlenmeyer flasks were used, each with 10,000 embryos resuspended at 1/μL in 10 mL. Flasks were put in a shaker at 180 rpm at 20°C, like was done for the time series samples. After 36 h of L1 arrest (48 h after bleach), the 36-h samples were collected by spinning down the 10 mL culture for each strain at 3,000 rpm, aspirating off excess S-basal, and then transferring the worms in ~100 μL to a 1.5 mL Eppendorf tube. The volume of the sample was further reduced by spinning again at 3,000 rpm and pipetting off excess liquid to bring the final volume down to approximately 10 μL. This small volume facilitates micro-scale RNA preparation. Samples were flash frozen in liquid nitrogen and stored at −80°C until RNA isolation. At 36 h, 100 μM auxin or ethanol was added to each of the remaining flasks. After 132 h of L1 arrest, auxin and ethanol samples were collected as described for the 36-h samples.

#### RNA isolation and library preparation for AMA-1 AID samples

RNA was isolated using TRIzol Reagent (Invitrogen# 15596026) following the manufacturer’s instructions with some exceptions noted below. The procedure was scaled down linearly, using 100 μL Trizol. 5 ug linear polyacrylamide (Sigma# 56575) was included as a neutral carrier for RNA precipitation. RNA was eluted in nuclease-free water and incubated at 55°C for approximately 4 min to resuspend the pellet. 25 ng of total RNA was used for each library preparation. ThermoFisher ERCC Spike-In Mix (Thermo #4456740) was added based on total RNA per manufacturer’s instructions. NEBNext Poly(A) mRNA Isolation Module (New England Biolabs# E7490) and NEBNext Ultra II RNA Library Prep kit (New England Biolabs# E7770) was used to perform poly-A selection and prepare libraries for sequencing respectively. The final libraries were enriched with 14 or 15 cycles of PCR, depending on batch. Libraries were then sequenced using Illumina NovaSeq 6000 to obtain 50 bp paired-end reads.

#### Differential expression analysis of AMA-1 AID RNA-seq data

Paired-end reads were mapped with bowtie using the following command: bowtie -I 0 -X 500 –chunkmbs 256 -k 1 -m 2 -S -p 2 using the WS273 version of the genome, with the sequences from the ERCC spike-ins appended to determine reads mapping to the spike-ins. Counts were determined as described for the time series analysis. Count data was imported into R, then filtered to include only protein-coding genes. To determine expressed genes, count data was further filtered to include only genes with counts >10 in at least four samples. The RUVseq package (Risso et al., 2014) was used to determine factors of unwanted variation prior to differential expression analysis. First, betweenLaneNormalization was used for upper-quartile normalization (which = ”upper”). Next, RUVg was used with k = 1 to improve normalization by normalizing based on the spike-ins. edgeR was then used for differential expression analysis, incorporating the factors of unwanted variation from RUVseq. glmFit and glmLRT commands (which fit a GLM and execute a likelihood ratio test in edgeR) were used to determine significant differences for pairs of conditions of interest.

#### Normalization with external spike-in standards

Counts mapping to spike-ins were plotted against the known concentrations of spike-ins in Mix 1 added to total RNA. A linear model was fit in R between the log_10_-normalized counts and log_10_-normalized concentration of each spike-in. Based on the slope and intercept values from the regression fit, all counts for non-spike-in genes were converted to units of attomoles per μL (the spike-in units). To convert to units of attomoles of transcript per worm, the normalized count data was then multiplied by the volume (in μL) of spike-in and divided by the average number of worms needed to acquire the amount of total RNA used for each condition.

#### Lysate preparation for western blot

Worm samples were collected as described for the mRNA-seq samples, with minor exceptions. Only the *Peft-3::TIR1; ama-1::AID* genetic background was used, and one of three biological replicates was collected with 25,000 instead of 10,000 worms for each condition (though density was held constant). Samples were stored at −80°C. The frozen pellet was rapidly freeze-thawed 3 times, cycling between liquid nitrogen and a 45°C water bath. Laemmli buffer (Sigma# S3401) was added to the samples to bring the final concentration to 1X. The samples were then incubated at 95°C for 10 min, frozen in dry ice for 15 min, and incubated at 95°C again for another 10 min. Debris was pelleted by centrifuging the samples at 14,000 rpm for 5 min, and the lysate was transferred to a new tube. Total protein was quantified using the Pierce 660nm Protein Assay kit (Thermo# PI22662) following the manufacturer’s instructions.

#### Western blot

Two μg of total protein per sample was loaded onto the NuPAGE 4%–12% Bis-Tris gel (Invitrogen# NP0321), along with 10 μL of Spectra Multicolor Protein Ladder (Thermo# 26634). Proteins were resolved at 200 V for 50 min in MOPS SDS running buffer. Following resolution, proteins were transferred to PVDF membrane (Invitrogen# LC2005) at 100 V for 60 min. The membrane was blocked in non-fat milk for 1 h to reduce non-specific binding. The blot was then probed with HRP conjugated anti-FLAG antibody (Sigma# A8592) at 1:2000 through overnight incubation at 4°C. The membrane was then washed multiple times with TBS buffer, supplemented with 0.1% Tween 20, to wash off excess unbound antibodies. SuperSignal West Femto Substrate (Thermo# 34095) was used to develop the blot, and it was imaged in the iBright FL1500 Imaging system (Thermo Fisher). A second NuPAGE gel, run with the same samples, was stained with SYPRO Ruby Protein Gel Stain (Invitrogen# S12001) following the manufacturer’s protocol and imaged to confirm equal loading.

### QUANTIFICATION AND STATISTICAL ANALYSES

Statistics were performed in R, and the statistical test used is indicated in figure legends. Linear mixed-effects models were used to control for the effect of biological replicate in cases in which there were multiple biological replicates and multiple observations originating from each replicate (*e.g*., Figures 4E and 4G). Biological replicate was considered a random effect, and the variable of interest was considered a fixed effect. A t test or one-way ANOVA was used in cases in which there was only 1 observation per replicate (*e.g*., Figures 4B and 4H). The Kolmogorov-Smirnov test was used to determine if there were differences in distributions of a groups of genes across conditions (*e.g*., Figures 5D and 5F).

## Supplementary Material

1

2

3

## Figures and Tables

**KEY RESOURCES TABLE:** 

REAGENT or RESOURCE	SOURCE	IDENTIFIER

Antibodies		

anti-FLAG antibody	Sigma	Cat# A8592; RRID:AB_439702

Bacterial and virus strains		

*Escherichia coli:* OP50	Caenorhabditis Genetics Center	WB ID: OP50

Chemicals, peptides, and recombinant proteins		

Indole-3-acetic-acid	ThermoFisher	A10556

Critical commercial assays		

NEBNext Ultra RNA Library Prep Kit	New England Biolabs	E7530
NEBNext Ultra II RNA Library Prep Kit	New England Biolabs	E7770
ERCC Spike-In Mix	ThermoFisher	4456740
TRIzol Reagent	Invitrogen	15596026
Linear Polyacrylamide	Sigma	56575
Laemmli buffer	Sigma	S3401
Pierce 660nm Protein Assay Kit	Thermo	PI22662
NuPAGE 4%–12% Bis-Tris gel	Invitrogen	NP0321
Spectra Multicolor Protein Ladder	Thermo	26634
PVDF Membrane	Invitrogen	LC2005
SuperSignal West Femto Substrate	Thermo	34095
SYPRO Ruby Protein Gel Stain	Invitrogen	S12001

Deposited data		

RNAseq data: starved L1 larvae 12-point time series	This paper	GSE173657
RNAseq data: starved L1 larvae with AMA-1 degraded in soma and germline and controls	This paper	GSE173657

Experimental models: Organisms/strains		

*C. elegans:* ieSi57 [*eff-3p*::TIR1::mRuby::*unc-54* 3’UTR + Cbr-*unc-119*(+)] II	Caenorhabditis Genetics Center	CA1200
*C. elegans:* ieSi57 II; ieSi58 [*eft-3p*::degron::GFP::*unc-54* 3’UTR + Cbr-*unc-119*(+)] IV	Caenorhabditis Genetics Center	CA1202
*C. elegans:* ieSi64 [*gld-1p*::TIR1::mRuby::*gld-1* 3’UTR + Cbr-*unc-119*(+)] II	Caenorhabditis Genetics Center	CA1352
*C. elegans: cec-4(ok3124)* IV	Caenorhabditis Genetics Center	RB2301
*C. elegans: ama-1(syb1513)*	This study	PHX1513
*C. elegans:* ieSi64 II; *ama-1(syb1513)* IV	This study	LRB387
*C. elegans:* ieSi57 II; *ama-1(syb1513)* IV	This study	LRB389

Software and algorithms		

Bowtie	Langmead (2010)	
edgeR	Robinson et al., (2010)	
HTSeq	Anders et al., (2015)	

## Highlights

- Gene expression changes for the duration of starvation-induced developmental arrest
- Somatic transcription is required early, but not late, to support starvation survival
- The germline remains transcriptionally quiescent, supporting future reproduction
- Germline transcripts are exceptionally stable compared to somatic transcripts
