# Supplementary material for: Alternative somatic and germline gene-regulatory strategies during starvation-induced developmental arrest
Source: Cell Rep. Author manuscript; Available in PMC 2022 Oct 27. (PMC9608353; doi:10.1016/j.celrep.2022.111473)
Supplement: 1 [file NIHMS1842083-supplement-1.pdf]

Cell Reports, Volume *41*

Supplemental information

Alternative somatic and germline gene-regulatory strategies during starvation-induced developmental arrest

Amy K. Webster, Rojin Chitrakar, Seth M. Taylor, and L. Ryan Baugh

SUPPLEMENTARY FIGURES

Supplementary Figure 1: Cluster analysis reveals dominant temporal patterns of gene expression in whole starved worms

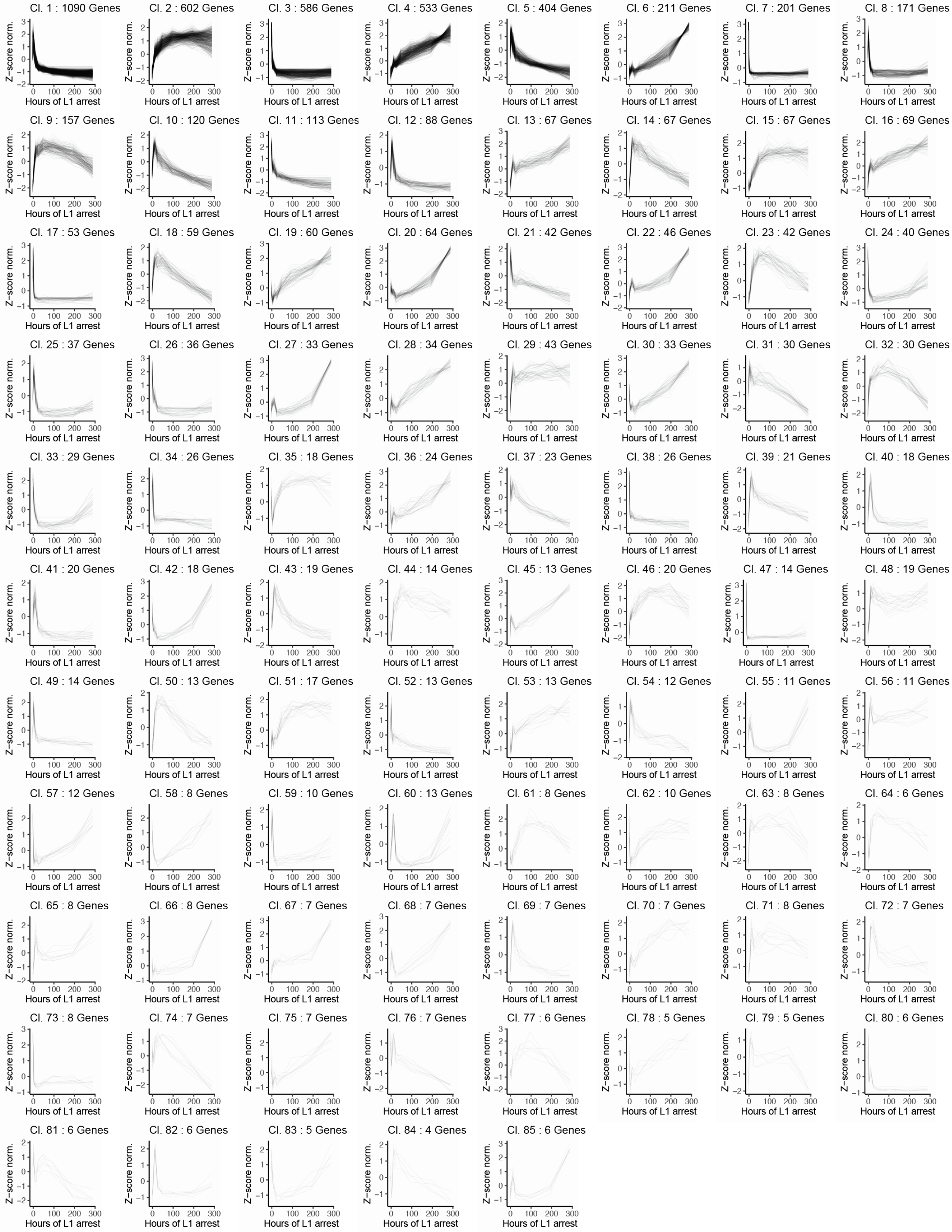

Supplementary Figure 1: Cluster analysis reveals dominant temporal patterns of gene expression in whole starved worms. Related to Figure 2. Z-scores over time for all genes included in Clusters 1-85 are shown, which includes all clusters with 5 or more genes. Clusters 86-129 are not shown, each of which has fewer than five genes.

Supplementary Figure 2: Temporal dynamics of additional transcriptional regulators

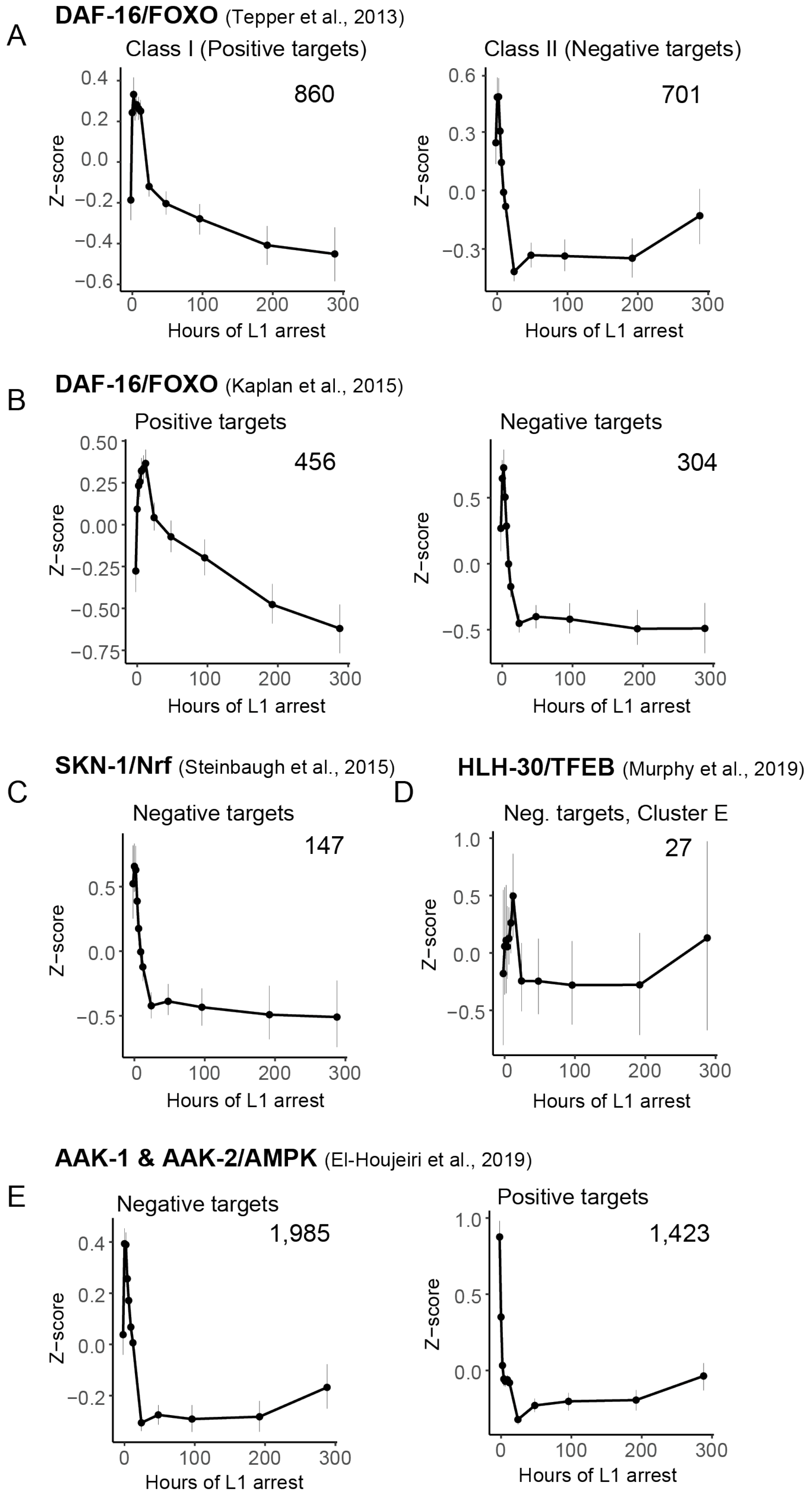

Supplementary Figure 2: Temporal activity dynamics of additional transcriptional regulators. Related to Figure 2. A-G. Average Z-scores over time for all genes included in clustering from the indicated regulator and cited dataset are plotted. Error bars show the 99% confidence interval surrounding the mean.

Supplementary Figure 3: Relative increase in germline gene expression is robust to increased stringency in defining germline genes

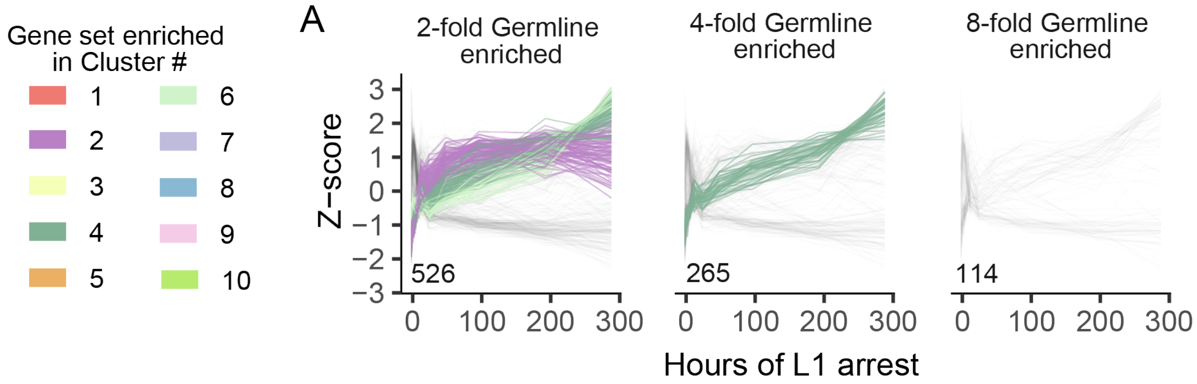

Supplementary Figure 3: Relative increase in germline gene expression is robust to increased stringency in defining germline genes. Related to Figure 3. A. Z-scores of all germline-enriched genes included in clustering are plotted over time. The number of genes plotted is indicated on the inset of each graph. Genes are color-coded by cluster if that cluster is enriched (hypergeometric $p < 0.05$) in the gene group.

Supplementary Figure 4: AMA-1 protein is degraded upon auxin addition during L1 arrest in *Peft-3::TIR1; ama-1::AID* background

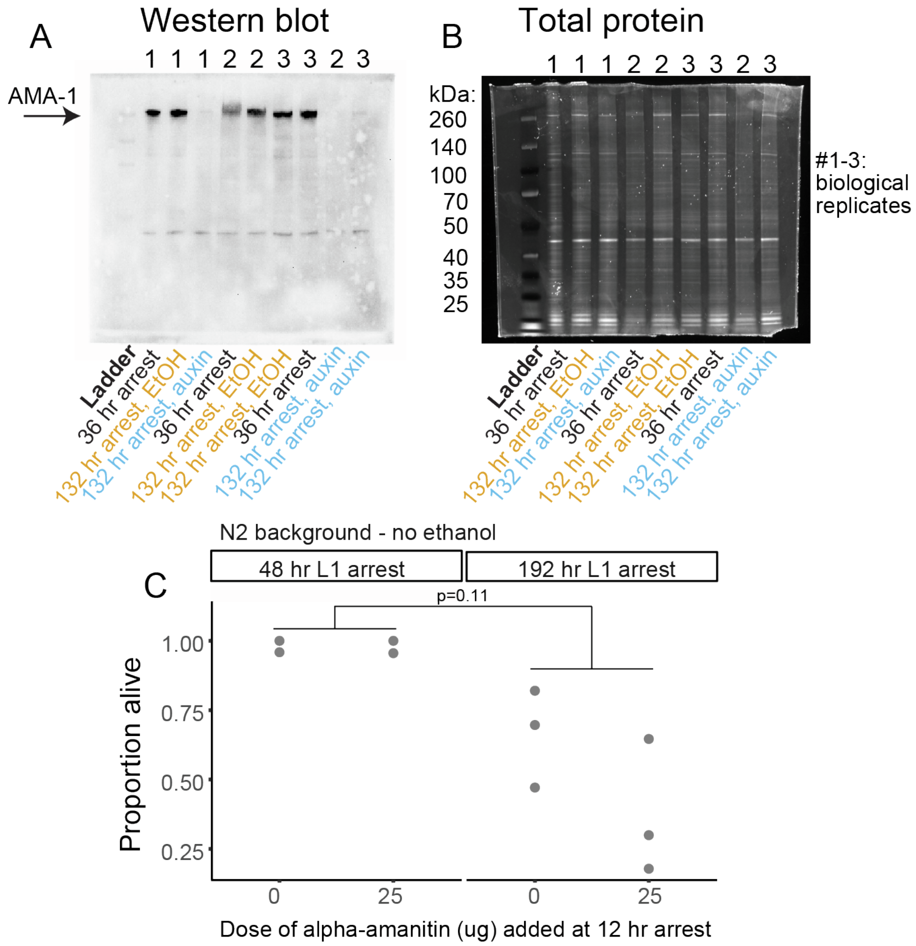

Supplementary Figure 4: AMA-1 protein is degraded upon auxin addition during L1 arrest in *Peft-3::TIR1; ama-1::AID* background. Related to Figure 4. A. Western blot probed with anti-FLAG to visualize AMA-1. Expected size of phosphorylated AMA-1 including the degron and 3xFLAG tag is ~218-258 kD. B. Total protein gel corresponding to Western blot in A. Equal amounts of total protein were loaded for all samples. A-B. The ladder and next three lanes are also shown in Figure 4D. C. N2 survival with and without addition of alpha-amanitin (Pol II inhibitor) addition. P-value calculated from linear mixed-effects model with interaction between time point and alpha-amanitin dose. Replicate was included as a random effect in the model.

Supplementary Figure 5: Survival, RNA content, and protein content in conditions used for *ama-1::AID* mRNA-seq

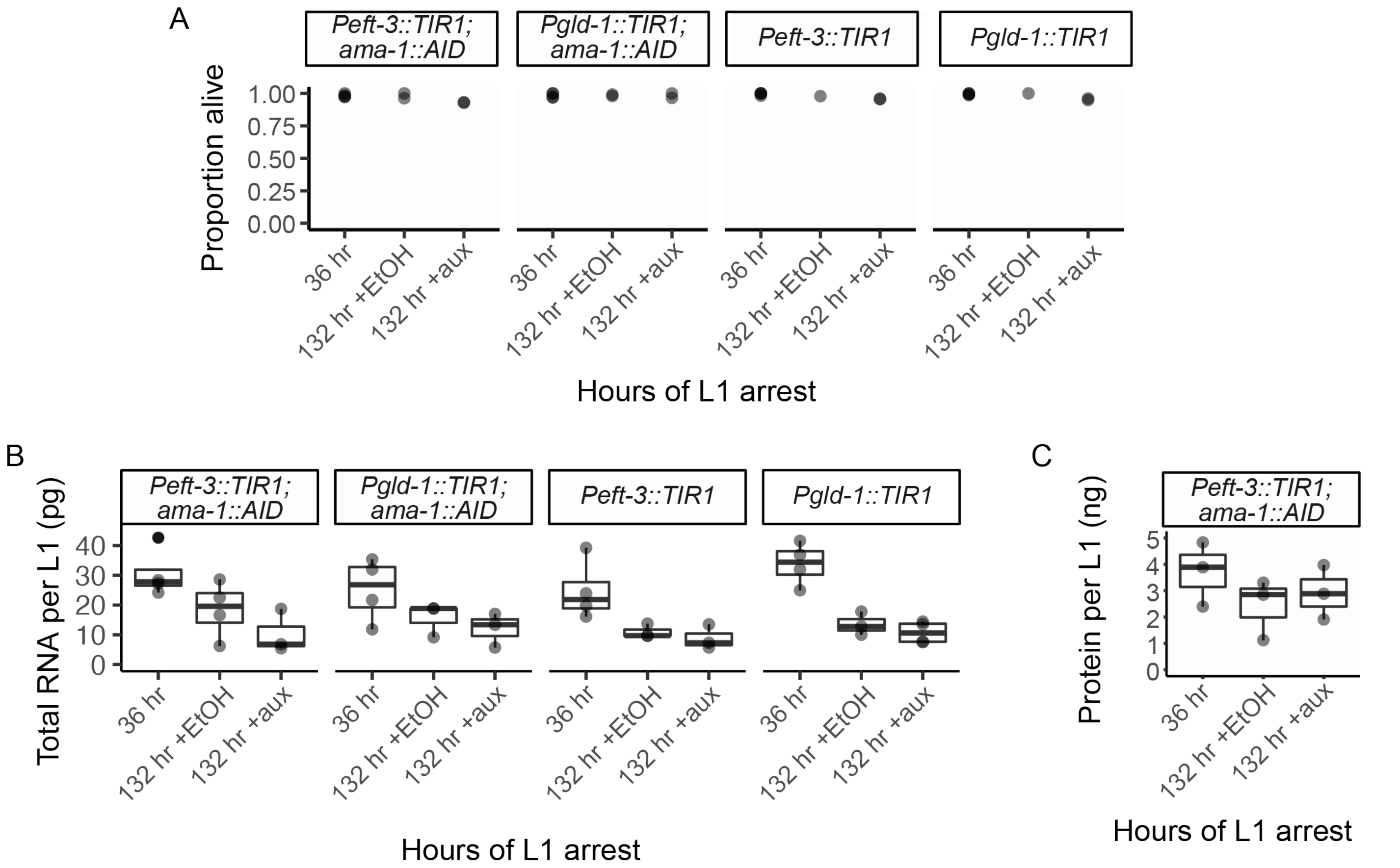

Supplementary Figure 5: Survival, RNA content, and protein content in conditions used for *ama-1::AID* mRNA-seq. Related to Figure 6. A. Proportion alive during L1 arrest at 36 hours, 132 hours with auxin, and 132 hours with ethanol. B. Total RNA content for all strains and conditions. One-way ANOVAs were performed within each time point across conditions and there were no significant differences. Data across strains was merged for Figure 6F. C. Total protein content across conditions in the *Peft-3::TIR1; ama-1::AID* background. There was no difference between 132 hours auxin and ethanol conditions in a t-test, so these were merged for Figure 6G.

Supplementary Figure 6: Known concentration of spike-ins vs. spike-in counts

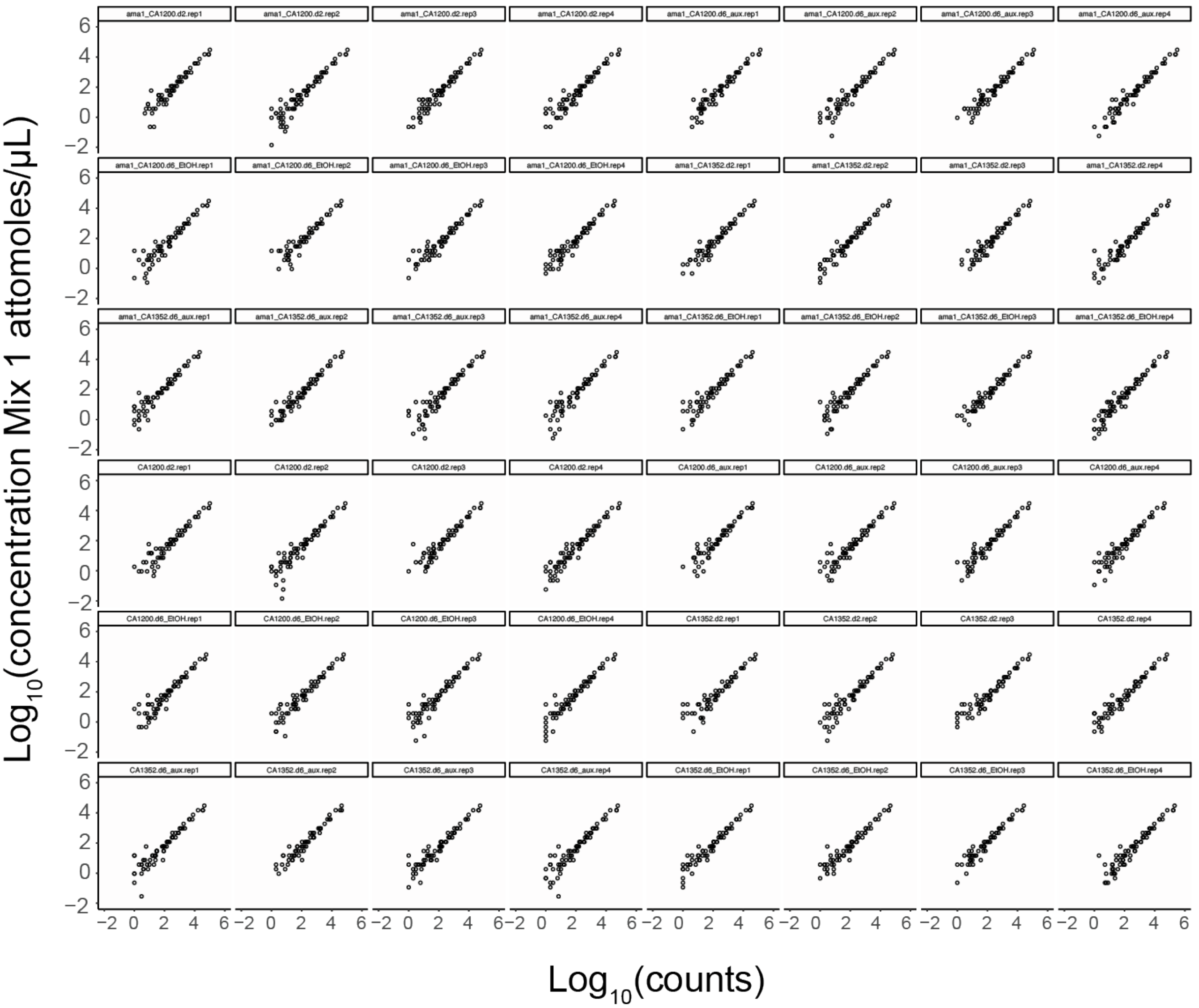

Supplementary Figure 6: Known concentration of spike-ins vs. spike-in counts. Related to Figure 6. mRNA-seq count data on the x-axis plotted against known concentration in attomoles per μL on the y-axis. A linear regression was fit to determine a normalization factor for each library for absolute normalization used for Figure 6H-I.
